# Crystal structure and cellular functions of uPAR dimer

**DOI:** 10.1038/s41467-022-29344-y

**Published:** 2022-03-29

**Authors:** Shujuan Yu, Yaqun Sui, Jiawei Wang, Yongdong Li, Hanlin Li, Yingping Cao, Liqing Chen, Longguang Jiang, Cai Yuan, Mingdong Huang

**Affiliations:** 1 College of Chemistry, Fuzhou University, Fuzhou, 350116 Fujian, China; 2 College of Biological Science and Engineering, Fuzhou University, 350116 Fuzhou, Fujian China; 3 Tsinghua University, 100084 Beijing, China; 4 Department of Clinical Laboratory, Fujian Medical University Union Hospital, 350001 Fuzhou, Fujian China; 5 School of Molecular Sciences, Arizona State University, 85287 Tempe, AZ USA; 6 Key Laboratory of Marine Enzyme Engineering, Fuzhou University, 350116 Fuzhou, Fujian China

**Keywords:** X-ray crystallography, Membrane proteins, Cell signalling

## Abstract

Receptor dimerization of urokinase-type plasminogen activator receptor (uPAR) was previously identified at protein level and on cell surface. Recently, a dimeric form of mouse uPAR isoform 2 was proposed to induce kidney disease. Here, we report the crystal structure of human uPAR dimer at 2.96 Å. The structure reveals enormous conformational changes of the dimer compared to the monomeric structure: D1 of uPAR opens up into a large expanded ring that captures a β-hairpin loop of a neighboring uPAR to form an expanded β-sheet, leading to an elongated, highly intertwined dimeric uPAR. Based on the structure, we identify E49P as a mutation promoting dimer formation. The mutation increases receptor binding to the amino terminal fragment of its primary ligand uPA, induces the receptor to distribute to the basal membrane, promotes cell proliferation, and alters cell morphology via β1 integrin signaling. These results reveal the structural basis for uPAR dimerization, its effect on cellular functions, and provide a basis to further study this multifunctional receptor.

## Abstract (web summary)

The structural basis for urokinase-type plasminogen activator receptor (uPAR) dimerization is not understood. Here, the authors solve the crystal structure of soluble uPAR dimers, identifying substantial structural changes compared to the monomer.

## Introduction

Urokinase-type plasminogen activator (uPA) receptor (uPAR) is a glycoprotein tethered to the cell membrane via glycosylphosphotidylinositol (GPI) anchor. uPAR mediates multiple physiological and pathologic processes^1,2^. In physiology, uPAR involves in the processes of tissue remodeling, wound healing, stress and immune response. In pathology, aberrant uPAR expression is frequently detected in organ failure^3–5^, cellular senescence^6^, auto-immune and infectious diseases^4,7^, and in solid tumors and several hematologic malignancies^8,9^. Soluble uPAR (suPAR), the ectodomains of uPAR, becomes a risk factor in a number of human clinical indications, including chronic and systemic inflammation^10^, focal segmental glomerulosclerosis (FSGS)^11,12^, acute kidney injury (AKI)^13,14^, chronic kidney disease (CKD)^15^ and diabetes^16,17^. The pleiotropic functions of uPAR are in alignment with uPAR capability to interact with an array of very different partners, including uPA, vitronectin, high molecular weight kininogen, GPCR, tyrosine kinase receptors, and a number of integrins^18–20^. For example, the interaction of uPAR with its primary ligand uPA^21^, a protease that efficiently activates plasminogen^22^, concentrates uPA proteolytic activity to the pericellular region to trigger degradation of fibrin and extracellular matrix. uPAR binds to the somatomedin B domain of vitronectin, integrins and receptor tyrosine kinases, and triggers intracellular signaling pathways.

Structural studies of uPAR, including suPAR in complex with its antagonist peptide^23^ and ligands (uPA^21,24^ and vitronectin^25^), showed that suPAR adopted a compact shape with its three globular domains (D1-D3) arranged in a triangular way, forming a central cavity that accommodates the epidermal growth factor domain of its ligand uPA or antagonist peptide. Another key ligand, vitronectin, binds at the outer side of the suPAR, near the boundary of D1 and D2^25^. This overall compact structural architecture of uPAR is preserved in murine suPAR:uPA complex^26^, in ligand-free uPAR in the presence of a unique anti-uPAR antibody (8B12)^27^, and in a uPAR mutant (H47C/N259C) with an engineered disulfide bond linking D1 and D3^28^. Despite these structural studies, it remains necessary to study how such a small receptor (283 amino acids) can recognize more than 42 binding partners^20^.

Recently, a dimeric form of mouse uPAR isoform 2 was proposed, which contains uPAR D1 and one-half of D2. The over-expression of this dimer induced kidney disease in mice and activated glomerular Src kinase via β_3_ integrin during the development of kidney disease^29^. Electron microscopy study of the recombinant isoform dimer revealed the globular nature of the structure but showed no molecular details due to limited resolution. uPAR dimerization was also previously identified at the protein level and on the cell surface^30–32^. Although uPAR dimerization is well defined, the structural basis of uPAR dimerization is unknown.

Here we reconstitute the soluble uPAR (suPAR) dimer in vitro, and show suPAR dimer has a stronger binding ability to amino terminal fragment of uPA (ATF) of uPA than monomer. We also determine the crystal structure of dimeric suPAR, which reveals the structural basis of uPAR dimerization and its structural flexibility. Based on the crystal structure, we find that the mutation of hinge residue Glu49 to proline promotes uPAR dimerization on the cell surface, demonstrating that the reconstituted suPAR dimer represents the dimer on the cell surface. Based on these results, we further find that uPAR dimerization promotes cell proliferation and alters cell morphology via β1 integrin signaling.

## Results

### Reconstitution of uPAR dimer in vitro

**Fig. 1:**
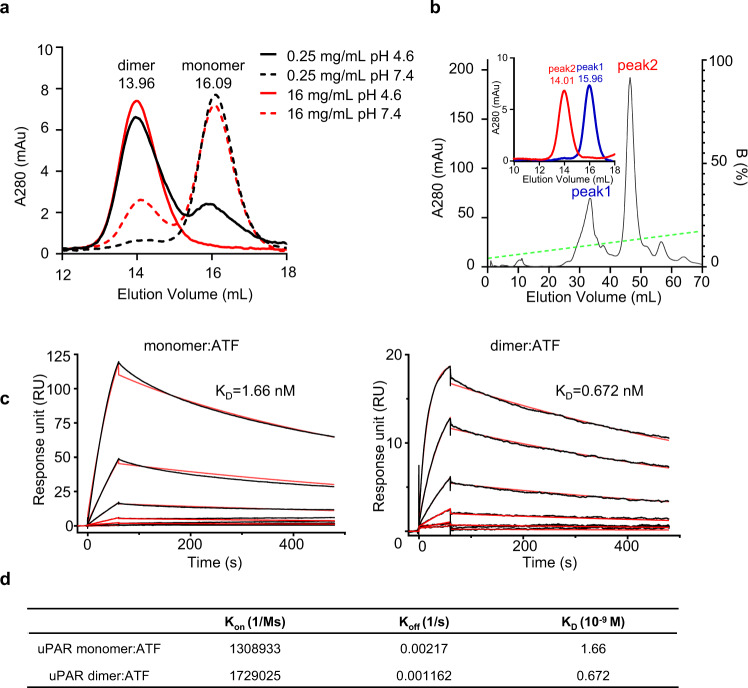
Reconstitution of suPAR dimer and its binding to ATF. **a** Recombinant suPAR formed into dimer at high concentration and low pH. suPAR was dissolved to 0.25 mg/mL concentration or 16 mg/mL concentration at low pH (pH 4.6) or neutral pH (pH 7.4) for 1 day, and was analyzed on a gel filtration column. **b** suPAR was separated into two peaks by ion-exchange chromatography, which was identified as monomer or dimer on gel filtration chromatography (insert). **c** Binding kinetics of the ATF, in 3-fold dilution, on suPAR monomer or dimer captured by anti-uPAR immobilized on CM5 chip by surface plasmon resonance. Sensorgrams recorded for the association (120 s), and dissociation (600 s) phases of the various ATF concentrations are shown as solid black lines with the corresponding curves fitting to a 1:1 Langmuir binding superimposed as solid red lines. The derived kinetic rate constants are shown in **d**. Source data are provided as a Source Data file.

Dimerization of GPI-anchored uPAR on the cell surface was first reported in 2002^30^ and further confirmed by different groups using various techniques, including chemical cross linking^32^, photon-counting histogram, fluorescence energy transfer^33,34^, and number and brightness image analysis^31^. However, the uPAR dimer has not been characterized yet. Here, we successfully reconstituted the uPAR dimer in vitro by using soluble uPAR lacking the GPI anchor. We found high protein concentration or low pH promoted dimer formation (Fig. 1a and Supplementary Fig. 1a). Once formed, this dimer was stable and would not dissociate into monomer even at low concentrations (to 3.5 μg/mL, Supplementary Fig. 1b). The dimer was separated out from the monomer by ion exchange (Fig. 1b). We next characterized the ligand-binding capability of the dimer. We found that dimeric suPAR bound to ATF of uPA^21^, vitronectin, high molecular weight kininogen, or streptococcal surface dehydrogenase based on a direct binding assay (Supplementary Fig. 2). These results demonstrated that dimeric suPAR maintained, to some extent, the ligand-binding profile of monomeric uPAR. Notably, suPAR dimer showed a stronger ATF-binding capability based on this assay. This result was further supported by the quantitatively surface plasmon resonance (SPR) measurements, where the ATF flowed through the monomeric or dimeric uPAR captured on an anti-uPAR-coated solid support, giving a dissociation constant of 1.66 nM and 0.672 nM, for the monomer and dimer, respectively (Fig. 1c). Full-length single-chain uPA (scuPA) was also found to bind to the monomer and dimer at comparable affinity (*K*_D_ of 0.149 and 0.141 nM, respectively, Supplementary Fig. 3). The quantitative result of SDS-PAGE of suPAR:ATF complex purified by gel filtration showed that one uPAR dimer bound to two ATF molecules (Supplementary Fig. 4).

### Crystal structure of dimeric suPAR

**Fig. 2:**
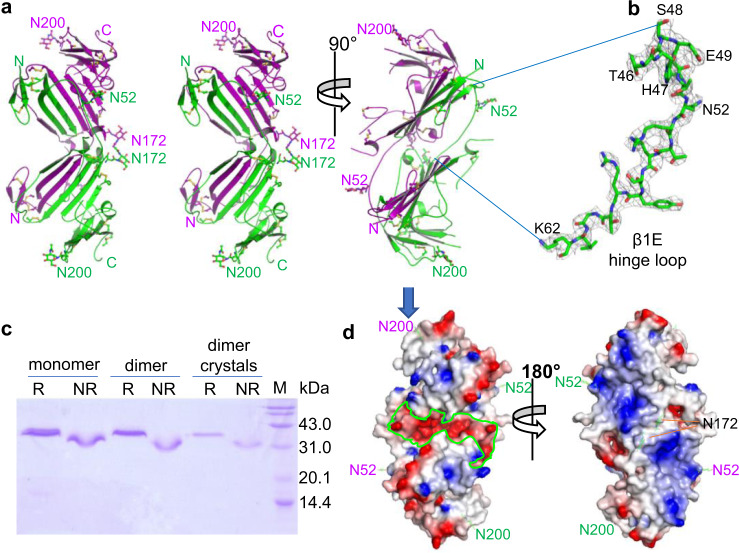
D1 swapping promotes uPAR dimerization. **a** Stereo ribbon representations (left two panels) and a perpendicular view (right panel) of dimeric uPAR, with each protomer colored as green and magenta, respectively. Four N-terminal β-strands (residues 1–48) of one suPAR merges into the other molecule as the swapped domain. Sugar moieties and disulfide bonds are shown as ball-and-sticks. **b** The domain 1 strand E (β1E) now becomes a hinge loop and links the swapped β-strands to the major domain (residues 63–277) of suPAR. The conformation of this hinge loop was verified by the 2Fo-Fc map electron density contoured at 1 sigma. **c** The SDS-PAGE of the crystals under reduced (R) or non-reduced (NR) conditions confirmed the presence of the intact full-length and non-covalently dimerized suPAR in the crystals. Source data are provided as a Source Data file. **d** Electrostatic surface representations of dimeric suPAR at two opposite faces (sugar moieties are shown in sticks and are labeled), viewed approximately along the 2-fold axis, showed a distinctly different charge distribution. The left panel was in the same orientation as in the right panel of **a** and showed the predominantly negatively charged groove (in bright green circle).

Crystallographic studies were carried out to reveal the molecular mechanism for uPAR dimerization. The crystal structure of dimeric suPAR under neutral pH shows that the two suPAR molecules intertwine tightly to each other, mainly through the exchange of the first four β-strands of the D1 of each protomer (Fig. 2a). The two suPAR protomers are symmetrically equivalent, linked by a 2-fold axis, and similar to each other (root mean square difference of 0.63 Å for main chain atoms). Most residues of dimeric suPAR are well-defined by electron density, including the hinge loop (Fig. 2b) and the long loop (residues 78–91) between D1 and D2 (Supplementary Fig. 5), which are proteolytic sensitive and often disordered in monomeric suPAR structures^21,23,25^. Human uPAR has five potential N-linked glycosylation sites, of which only four are observed actually to be glycosylated^35,36^. Our dimeric suPAR structure demonstrates that these glycans point away from the protein surface and are not involved in the domain assembly, and are fully compatible with previously reported glycosylation pattern^35,36^, further validating the current dimeric suPAR structure. A large segment of the D2 (mainly residues 100–114, 124–146, the first two fingers) is disordered and not visible in the current structure. While the SDS-PAGE of the crystals confirmed the presence of the intact full-length and non-covalently dimeric suPAR (Fig. 2c).

The dimeric suPAR appears as a rod-shaped molecule with a large groove in the middle, and has a molecular dimension of 40 × 60 × 95 Å (excluding the carbohydrate moieties and missing residues). There are extensive interactions between these two suPAR protomers, with approximately 3799.4 Å^2^ surface area buried upon dimerization. This buried surface area accounts for ~24% of the total surface of each protomer, and involves extensive polar interactions (76 hydrogen bonds and 11 salt bridges, Supplementary Tables 1 and 2) between the two protomers, especially in swapping D1 domain (Supplementary Fig. 6). The dimeric suPAR has a distinctly different charge distribution on its two opposite faces (Fig. 2d). In one face (left panel in Fig. 2d), the central groove is highly negatively charged (colored red), while another face (right panel in Fig. 2d) is lined by positively charged residues (colored blue).

### Comparison of the dimeric and monomeric structures of suPAR

The structure of dimeric suPAR is very different from ligand-bound monomeric suPAR. In the dimeric suPAR, the carboxy segment of uPAR (D3 and C-terminal region of the D2) has conformations similar to the ligand-bound monomeric suPAR (with a r.m.s. deviation of 1.2 Å for the Cα atoms of residues 152–277, Supplementary Fig. 7a). Dramatic differences are observed in the amino segment of uPAR (D1 and N-terminal part of D2).

**Fig. 3:**
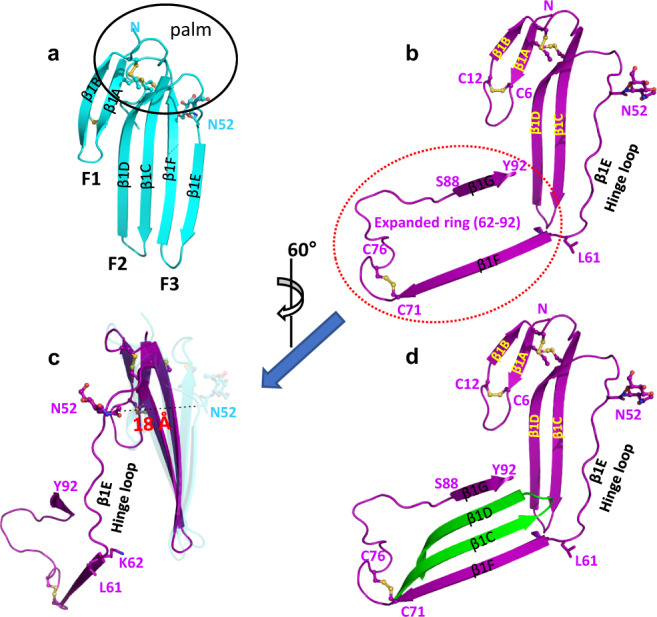
Major conformational changes of the D1 of dimeric suPAR (this work) compared to the ligand-bound suPAR (PDB 3BT2). **a** D1 in the ligand-bound suPAR (cyan) forms a typical three-fingered fold with a palm region (circled) packed with highly conserved disulfide bridges. The three fingers of this fold are labeled F1-F3. **b** In dimeric suPAR, the D1 of one protomer (magenta, in an orientation similar to in **a**) has only two fingers with the β1F strand of the third finger swings away from the D1 pivoting on a hinge (residue 60–62, black dashed circle) of D1. The β1F strand, along with a long loop between the D1 and D2, forms a long expanded ring (residue 62–92, circled in the red dashed circle). **c** A 60°rotation along Y axis of the B orientation shows the β1E in D1 of dimeric suPAR (magenta) dislodges away, in parallel, from the first and the second fingers, leading to a shift of 18 Å, compared to the D1 of ligand-bound suPAR (shown in transparent cyan). **d** The expanded ring captures the β1C- β1D hairpin (green) of the other protomer of suPAR. Such interactions are the key for dimeric suPAR formation.

In the uPAR:ATF structure, the D1 adopts a typical three-fingered fold of LU domain, consisting of a disulfide-bond-rich palm region^21,23^ and three fingers containing two β strands per finger (Fig. 3a). The β strands of these three fingers are adjacent to each other, and together, form an extended β sheet (Fig. 3a). In dimeric suPAR, the D1 opens up with a large positional dislocation of its third finger (strands β1E-β1F) (Fig. 3b). The β1E dislodges away from the first and the second fingers perpendicular to the β sheet of D1. This leads to a shift of 18 Å of the β1E from its original position (Fig. 3c), and a shift of 26 Å of the carbohydrate moiety at the β1E (residue 52). The β1F moves away from the β1E, pivoting on the segment of Gly60-Lys62 (Figs. 2b and 3b). A proteolytic sensitive loop of uPAR, which is typically disordered in monomeric suPAR, is now ordered in the dimer structure. This loop, together with the β1F strand, forms a large expanded ring with a length of 30 residues (residues 62–92, Fig. 3b). The expanded ring captures a finger from the neighboring suPAR protomer (the β1C-β1D hairpin, Fig. 3d) and forms a β sheet. Thus, the D1 is the primary driving force for the formation of dimeric uPAR, and provides most of the stabilizing energy (Supplementary Fig. 6).

Another distinguishing feature of the dimeric suPAR, compared to monomeric suPAR, is the disordering of the N-terminal part of the D2 (residues 101–116, 124–150). In addition, the disulfide bonds of D2 undergo drastic spatial rearrangement (Supplementary Fig. 7b). This is quite unusual because the LU domain of uPAR, named the three-fingered protein fold, contains four conserved disulfide bonds, and such disulfide bonding pattern is highly conserved^37^. This observation demonstrates that the cysteine-rich three-fingered fold has large conformational dynamics, despite the presence of multiple disulfide bonds.

### Mutations of the hinge residue E49 to proline promoted uPAR dimerization on cell surface

To validate the uPAR dimer structure, we generated a panel of uPAR mutants. Our structure identifies that the β1E converted from a β strand to the hinge loop (Fig. 2b) as the critical structural transition when compared uPAR dimer with the monomer. In this hinge loop, the residues 47–49, located next to β1D, appear to be the key hinge residues for uPAR dimer domain swapping. Previous studies on other domain swapped proteins showed that shortening or substituting the hinge region may favor one hinge conformation over the other^38,39^. We mutated these hinge residues to proline, phenylalanine, or alanine to study their roles for uPAR dimerization. uPAR^T51C/V70C^ mutant was also generated to disrupt the dimerization by restricting the movement of β1F away from β1E. Another mutant (uPAR^H47C/N259C^) that locked uPAR in the monomeric conformation identical to suPAR^28^ was used as a control. mRuby3, a type of fluorescent protein, was inserted between the extracellular C-terminal of uPAR and the GPI-anchoring sequence to study the location and expression level of uPAR on the cell surface. This form of chimera was previously reported and did not disrupt the function of uPAR^32^.

**Fig. 4:**
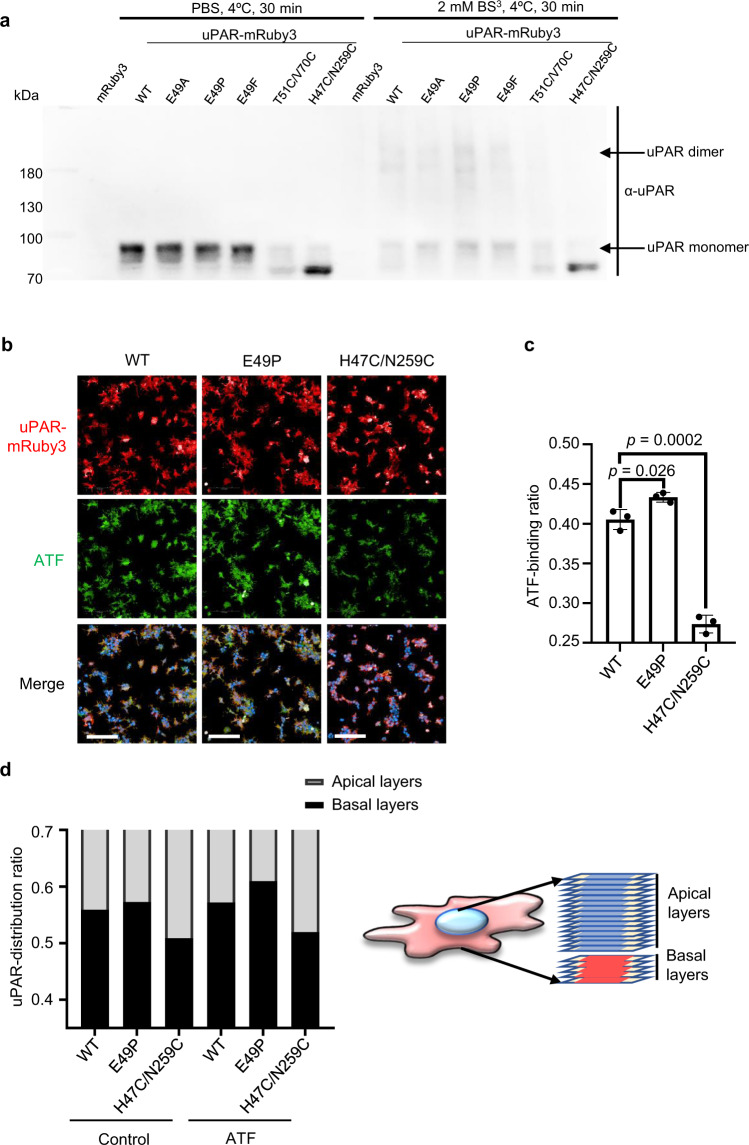
Mutations of the hinge residue Glu49 promoted uPAR dimerization on cell surface. **a** Anti-uPAR immunoblotting of mRuby3-tagged uPAR mutants stably expressed in 293T cells. The cells were treated with the chemical cross linker BS3, washed, and lysed. Cell lysates were separated and analyzed by immunoblotting using a polyclonal anti-uPAR antibody (α-uPAR). Source data are provided as a Source Data file. **b** Microscopic images of uPAR-mRuby3 expressing 293 T cells treated with FITC-ATF (green) for 1 h and Hoechst (blue) for 10 min before fixation. Scale bars, 200 µm. **c** Quantification of ATF-binding ratio of **b**. ATF-binding ratio was measured by comparing the total intensity of FITC to the summed intensity of mRuby3 in each well. The summed intensity of FITC and mRuby3 were calculated by Operetta CLS software. Data were representative of three independent experiments. Data are presented as mean ± SD and the *p*-values of two-tailed unpaired Student’s *t* test are indicated. Source data are provided as a Source Data file. **d** uPAR dimerization changed uPAR membrane distribution. The uPAR distribution ratio was measured based on the mRuby3 intensity of apical layers (15 layers) and basal layers (5 layers) divided by the mRuby3 intensity of the whole cell (illustrated on the right) and analyzed by Operetta CLS software. These 20 layers covered 10 μm total vertical distance from the basal membrane. Source data are provided as a Source Data file.

We expressed these mRuby3-fused mutants of uPAR together with a Flag-tagged wild-type uPAR in 293T cells in order to form dimeric uPAR, followed by pull-down using anti-Flag beads and probed with anti-uPAR antibody. We found that Flag-tagged uPAR had a significantly higher affinity to uPAR^E49P^ than to any other mutants (Supplementary Fig. 8a). We then constructed stable 293 T cell lines overexpressing mRuby3 fused with the hinge mutants of uPAR (E49), uPAR^T51C/V70C^ mutant, or uPAR^H47C/N259C^ mutant by infecting lentivirus. We treated these uPAR mutants expressed on 293T cells with membrane-impermeable chemical cross linker BS3, and followed by an immunoblotting assay using anti-uPAR. The results showed that the uPAR^E49P^ 293T cells contained more dimeric uPAR than uPAR^WT^ 293T cells on the cell surface, while no dimeric uPAR on the uPAR^H47C/N259C^ 293T cells and few on uPAR^T51C/V70C^ 293T cells were found (Fig. 4a). These results demonstrated that E49 was critical for the dimer formation, and the E49P appeared to lock the hinge in a conformation preferred for the dimer. Thus, we chose uPAR^WT^, uPAR^E49P^ and uPAR^H47C/N259C^ 293T cells as the objects for further research.

Next, we detected the uptake of FITC conjugated ATF by the cells expressing different uPAR mutants. We found that the probe was uptaken more on uPAR^E49P^ 293T cells, either at 1 h or 6 h, than any other mutants (Fig. 4b, c, and Supplementary Fig. 8b, c), which was consistent with the in vitro results where dimeric uPAR showed stronger binding to ATF than monomeric uPAR (Fig. 1c, d).

We examined the cellular distribution of uPAR by imaging 20 layers (total 10 μm) of mRuby3 signal starting from basal membrane for the mRuby3 fused uPAR and its mutant expressed on 293T cells by high content imager. We found that uPAR^WT^ was asymmetrically distributed and accumulated more at the basal membrane. The uPAR^E49P^ mutant had more accumulation on the basal membrane than wild-type uPAR. On the contrary, the distribution of uPAR^H47C/N259C^ was homogeneous over the whole cells (Fig. 4d and Supplementary Movie 1–3). The incubation with ATF for 6 h further increased the accumulation of both uPAR^WT^ and uPAR^E49P^ on the basal membrane, but did not affect the distribution of uPAR^H47C/N259C^ (Fig. 4d and Supplementary Movie 4–6). These results are consistent with the previous report that uPAR dimers accumulated in patches at the basal membrane^33^.

### uPAR dimer and monomer showed distinct functions on cell proliferation and cell morphology

We observed clear differences in cell proliferation and cell morphology among these uPAR overexpressing cell lines. We next sought to delineate the roles of uPAR dimer and monomer on those cellular functions. The 293T cell lines overexpressing uPAR^E49P^ or uPAR^H47C/N259C^ mimicked uPAR dimer or monomer, respectively, based on our structural and functional study.

**Fig. 5:**
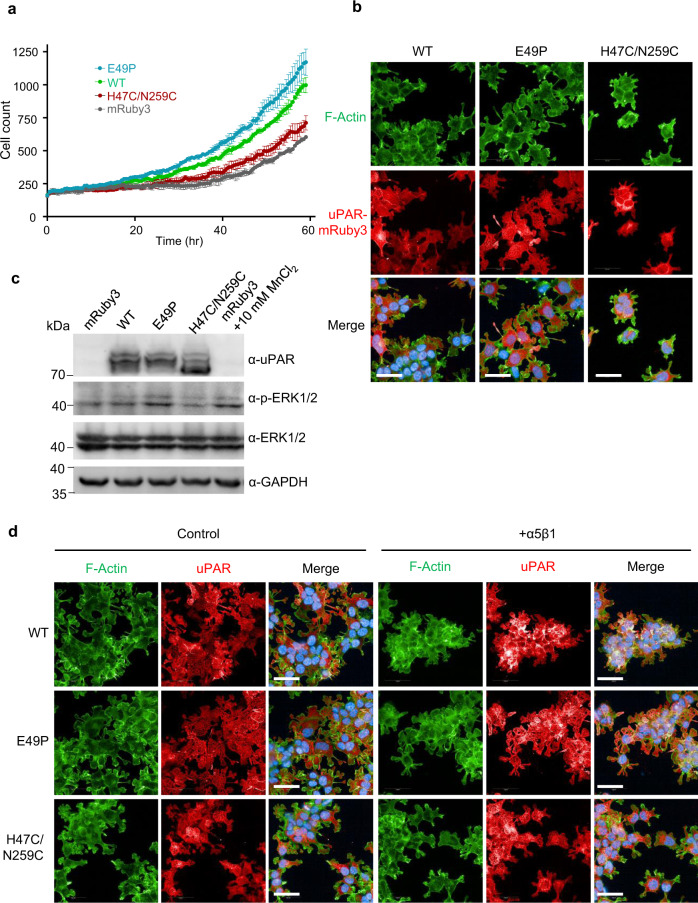
uPAR dimer had a stronger impact than monomer on cell proliferation and cell morphology. **a** Cell proliferation curve was generated by analyzing images of DPC (digital phase contrast) for different cell lines as indicated using Operetta CLS software. The DPC channel was recorded every half an hour for 60 h on HCS in the cell culture condition (37 °C, 5%CO_2_). Data are presented as mean ± SD. Source data are provided as a Source Data file. **b** Microscopic images of the cytoskeleton (green) of uPAR mutants fused with mRuby3 (red) stable cell lines. The cells were treated with Hoechst (blue) for 10 min before fixation and incubated with Actin-Tracker (green) for 1 h after 10-min permeabilization with 1% Triton X-100. Scale bars, 50 µm. **c** Immuno-analysis of mRuby3-tagged uPAR mutants stably expressed in 293T cells. The mRuby3 293T cells were treated with 10 mM MnCl_2_ for 30 min as a positive control. Cell lysates were separated and analyzed by immunoblotting using polyclonal anti-uPAR antibody (α-uPAR), anti-ERK1/2 (α-ERK1/2), anti-phospho-ERK1/2 (α-p-ERK1/2), and anti-GAPDH (α-GAPDH). Source data are provided as a Source Data file. **d** Microscopic images of the cytoskeleton (green) of uPAR mutants fused with mRuby3 (red) stable cell lines treated with integrin for 3 h, then stained as **b**. Scale bars, 50 µm.

uPAR^E49P^ cells grew faster than uPAR^WT^ cells, while the uPAR^H47C/N259C^ cells grew slower than uPAR^WT^ cells, based on imaging on high content imager (Fig. 5a). This result was further confirmed by using a different assay - CCK-8 assay (Supplementary Fig. 9). uPAR^H47C/N259C^ cells showed similar proliferation ability as the control mRuby3 cells, while uPAR^E49P^ and uPAR^WT^ cells showed faster cell proliferation. Both results demonstrated that dimerization of uPAR on cell surface promoted cell proliferation. Morphologically, uPAR^E49P^ 293T cells showed more extensive lamellipodia than uPAR^WT^ cells. In contrast, uPAR^H47C/N259C^ cells showed more cell–cell contacts, more membrane ruffles, and few extensive lamellipodia as well as a distinctive F-actin cytoskeletal reorganization, when compared to the uPAR^WT^ 293T cells, as observed by fluorescence microscopy after cell permeabilization and FITC-phalloidin (Actin-Tracker Green) staining (Fig. 5b).

As a GPI-anchored protein without cytoplasmic effector domain, uPAR was thought to cooperate with multiple transmembrane proteins, e.g., integrins, to mediate signal transduction^40,41^. Integrin β1, rather than β3 integrin, dominates in uPAR-overexpressed HEK293 cells^42,43^. uPAR was also reported to activate α5β1 integrin and ERK signaling, inducing in vivo proliferation of some human carcinomas^43,44^. We suspected integrin β1 signaling might participate in uPAR dimerization mediated cell proliferation and morphology change. To evaluate this hypothesis, we then examined ERK activation among these cell lines by western blotting. We found that uPAR^E49P^ cells had the strongest ERK1/2 phosphorylation signal, comparable to MnCl_2_ activated integrin signal in mRuby3 cells, while uPAR^H47C/N259C^ showed the weakest ERK1/2 phosphorylation signal (Fig. 5c). Moreover, when treated with recombinant soluble integrin α5β1 to compete for the interaction of uPAR:integrin, we observed that such treatment rendered both uPAR^E49P^ and uPAR^WT^ cells with stronger cell clustering by increasing cell–cell contacts, and had more membrane ruffles, as well as fewer lamellipodia, similar to uPAR^H47C/N259C^ cells with/without recombinant soluble integrin α5β1 treatment (Fig. 5d). These results indicated that the uPAR dimer promoted cell proliferation and altered cell morphology via β1 integrin signaling.

## Discussion

The presence of uPAR in dimer^30^ or aggregate^45^ forms was observed in the early days. Recently, mouse uPAR isoform 2 was found to exist in dimeric form and proposed to activate glomerular Src kinase via β3 integrin in the development of kidney disease^29^. Our current study demonstrated dimerization of uPAR changed its cell surface distribution, altered cell morphology, and promoted cell proliferation. Our studies indicated that uPAR dimer and monomer had distinct functions.

**Fig. 6:**
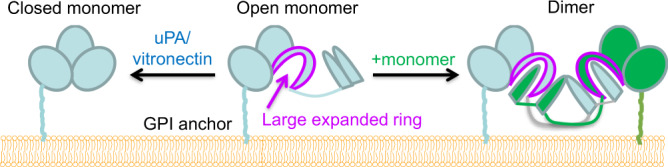
A schematic model of uPAR dimerization on the cell surface. The unbound uPAR is in dynamic equilibrium between the close (left) and open (middle) conformations. The presence of ligand uPA or vitronectin stabilizes the close conformation (left). A key feature in the open conformation is that uPAR forms a large expanded ring (residues 62–92, colored in magenta). When concentrated on the lipid-raft area of the cell surface, the large expanded ring can capture one β-hairpin loop of another uPAR to form a dimer (right panel) or even oligomer.

A schematic model of uPAR dimerization on the cell surface (Fig. 6) was generated, based on the dimeric suPAR structure and previous biochemical studies^46–48^. This is to highlight a hallmark structural feature of this dimeric suPAR structure: its two fingers (β1E and β1F) of D1 moves away from the rest of the suPAR, while β1F, along with the long loop between the D1 and D2, forms an expanded long ring (30 residues, Fig. 3b). This expanded ring then binds to a β-hairpin finger of another suPAR (Fig. 3d).

Dimerization is also found in other GPI-anchored proteins to regulate cellular functions or to modify cellular distribution. For example, CD59 forms transient homodimers in resting cells, which develops into the stable cluster (up to 4 molecules) upon ligand binding, suggesting that transient oligomer as platforms for transducing extracellular signals^49^. Dimerization of prion protein is also shown as an important molecular switch for both intracellular signaling and inactivation by releasing prion protein N-terminal domain or shedding^50^.

Monomeric uPAR exposes a large hydrophobic patch in its central pocket, which is sequestered in the presence of its ligand ATF, leading to a stabilized complex^21^. We proposed that in the absence of the ligand, uPAR would undergo conformational changes to sequester its hydrophobic patch from the aqueous environment^51^. Such conformational changes are indeed possible due to the conformational flexibility of uPAR. The current dimeric suPAR structure shows that the D2 and the third finger (β1E-β1F) of the D1 are the main sources of structural flexibility. A study demonstrated the structural flexibility of the third finger (β1E-β1F) of the D1 based on the hydrogen/deuterium exchange of suPAR^48^. The study also showed the D1-D2 boundary could be quite flexible from SAXS measurements^48^. In another study, a monoclonal antibody (Mab IIIF10) that recognized the β1E strand (residue 52–60) of uPAR could not bind to uPAR on U937 cells in the presence of a saturated amount of uPA^46^, but did so in the absence of ligand (uPA), consistent with the observation that this strand was buried in the uPAR:uPA complex^21^. These results consistently demonstrate that the flexibility of uPAR in the absence of ligand ATF is mainly due to the third finger (β1E-β1F) of the D1, and the uPA/ATF ligand stabilizes the conformation of the D1. It should be pointed out that another uPAR ligand, vitronectin, is also likely to reduce the flexibility of the third finger (β1E-β1F) of the D1 and stabilize the uPAR D1 conformation. This is because vitronectin binds at the β-turn connecting the β1E-β1F strands as well as the D1–D2 boundary^25^. The third finger of D1 also participates in the construction of uPA binding cavity, and its residues (L55, I63, I66) contribute to the hydrophobicity of the binding cavity.

The D1 of uPAR is exceptional as it lacks the third conserved disulfide bond in the palm region of the three-fingered protein fold, which is commonly existed in many three-fingered fold domains, including uPAR D2 and D3^37,52^. The absence of the restrain imposed by this disulfide bond allows its third finger to adopt an open conformation and account for the flexibility of the third finger of the D1. This lack of the third disulfide bond in the D1 is conserved in uPAR of other species based on sequence alignment of the uPAR from different species (Supplementary Fig. 10). The glycosylation patterns of uPAR are also genetically conserved. The hinge residues 47–49 in human uPAR, which is important for dimeric formation, are also conserved among different species. Thus, uPAR in other species may also demonstrate flexibility and may form dimer.

uPAR involves diverse physiological and pathological processes by interaction with different ligands or co-receptors. The diverse ligands or co-receptors binding capability may be due to the multiple conformations and forms of uPAR in vivo. Our current structure and the previous studies^21,25,48^ reveal the structural flexibility of uPAR. This flexible structure allows it to adjust its conformation upon ligand/co-receptor binding. One obvious example is uPA-induced conformational change endowed with its high affinity for vitronectin^53^. Additionally, the peptide in the so-called ligand-unloading loop (residue 130–140)^28^ was reported to mediate uPAR interaction with integrins, including integrin α5β1, αVβ3^54^ and αVβ6^55^. The conformation change of the peptide upon ligand binding may affect its interaction with these integrins. Also, in our current structure, the largely expanded ring of uPAR can capture one β-hairpin loop of another uPAR to form a dimer or even oligomer (Fig. 3). Besides forming a dimer, this large expanded ring can also interact with other molecules containing a β-hairpin to form a hetero-dimer. Besides, there are various forms of uPAR in vivo. The generation of these uPAR variants involves shedding suPAR from the cell surface by cleavage at the glycolipid anchor, and cleavage of uPAR/suPAR in the linker between D1 and D2 by uPA and MMPs to generate D1 and D2D3^56^. These uPAR variants display different functions. Only the full-length uPAR or suPAR can efficiently bind with uPA, and promote ECM degradation^21,57^. The fragment D2D3 of uPAR/suPAR, but not full-length uPAR/suPAR, could bind to formyl peptide receptors (FPRs) and function as a chemotactic agent for promoting the immune response^58^. uPAR structural diversity and flexibility determine its fitness to multiligand, which further specifies its multifunction.

## Methods

### Materials

Integrin α5β1 protein (CT014-H2508H) was purchased from SinoBiological. Urea-treated vitronectin was obtained from Molecular Innovations, MI, USA. High molecular weight kininogen (HMWK) was obtained from Enzyme Research. Streptococcal surface dehydrogenase (SDH) was expressed in *E.coli* BL21 (DE3) strain and purified by Ni-NTA. Anti-uPAR antibody ATN658 was a kind gift from Dr. Andrew Mazar of Monopar Therapeutics Inc. Plasmid pcDNA3.1-suPAR-mRuby3-GPI was constructed by Sangon Biotech. Oligo nucleotides were synthesized by Sunya Biotech (Supplementary Table 3). Horseradish peroxidase-conjugated antibody to rabbit IgG (S0101) or to mouse IgG (S0100) and anti-GAPDH (G0100) were purchased from LabLead; anti-phospho-ERK1/2 (p-ERK1/2) (#4370) was purchased from Cell Signaling Technology; anti-ERK1/2 (T55487) was from Abmart. Polyclonal rabbit anti-human uPAR antibody was prepared by Zoonbio biotechnology company using our recombinant soluble uPAR as antigen.

### Cell culture

*Drosophila* S2 cells were purchased from Invitrogen and cultured in EX-CELL® 420 Serum-Free Medium (Sigma). HEK293T (or 293 T) cells from ATCC were confirmed to be negative for mycoplasma contamination. 293T cells were cultured at 37 °C with 5% CO_2_ in Dulbecco’s Modified Eagle medium (DMEM) supplemented with 10% FBS (Gibco) and 1X penicillin-streptomycin (Invitrogen).

### Recombinant suPAR expression and the purification

Recombinant suPAR (residue 1–277) were secreted from *Drosophila* S2 cells as previously described^59^. The secreted protein was captured from the conditioned medium using an ATF affinity column followed by reversed-phase HPLC using a C4 column on the HPLC system. The fractions containing the pure protein were lyophilized.

To produce the selenomethionine (SeMet)-substituted suPAR, stable suPAR S2 cells were grown to 2L and reached a concentration of 10–12 × 10^6^ cells/mL in EX-CELL 420 serum-free medium. The cells were then washed in a methionine-free medium (Orbigen) and starved in this medium for 4–6 h at 28 °C before 60 μg/mL selenomethionine (Acros Organics) was added to the culture. The protein expression was induced with the addition of 500 μM of CuSO_4_. The culture medium was harvested 4–5 days after induction. The purification was similar to that of the native protein. Amino acid analysis showed that 90% of native methionine in the protein had been substituted by SeMet.

### suPAR dimerization

To search for the condition of the uPAR dimer, we dissolved the powder with buffers under pH 4.6 and pH 7.4 at different concentrations (0.25 mg/mL, 1 mg/mL, 4 mg/mL and 16 mg/mL). After being incubated at 4 °C for one day, the samples were analyzed on a gel filtration column (Superdex 200 10/300) with a buffer of 20 mM Tris pH 7.4, 150 mM KCl, at flow rates of 0.5 mL/minute. Elution profiles were monitored at 280 nm.

### Purification of dimeric suPAR

For suPAR crystallization, the lyophilized powder was dissolved in buffer under pH 4.6 at a concentration of 30 mg/mL. After being incubated at 4 °C for two days, the sample was adjusted to pH 8.0 with 1 M Tris pH 8.5, followed by purification on a ResourceQ column with a linear gradient of 0.08–0.3 M sodium chloride in 20 mM Tris pH 8.0. The dimeric suPAR was well separated from the monomer under this chromatographic condition and was concentrated and stored at −80 °C.

### Recombinant ATF expression and the purification

ATF was expressed and purified as described previously^60^. Briefly, after initial cultivation, the transformed *Pichia pastoris* strain X-33 cells in BMMY medium (1% yeast extract, 2% peptone, 100 mM potassium phosphate, pH 6.0, 1% v/v methanol) were supplemented daily with methanol (at a final concentration of 1%) over 4 days to induce the expression of ATF. The supernatants were collected, filtered, diluted with equal volume of H_2_O, and applied to fast flow cation exchange column (SPFF) for the capture of target proteins. The captured protein was eluted by potassium phosphate buffer containing 0.5 M NaCl, pH 6.5, and further purified on a gel filtration column Superdex75 HR 10/30 with 20 mM potassium phosphate buffer containing 0.15 M NaCl, pH 6.5. The fraction containing ATF from Superdex75 was collected and concentrated for further use.

### Recombinant scuPA expression and the purification

Recombinant single-chain uPA was secreted by *Drosophila* S2 cells and purified by SPFF. The S2 culture supernatant containing scuPA was collected, filtered and applied on SPFF column equilibrated in 50 mM Bicine buffer pH 8.0 and was eluted with a 0–0.5 M NaCl gradient. The fraction containing scuPA was concentrated using a Millipore YM-3 membrane and purified using a semi-preparative C8 reverse phase column (10 × 25 cm) on HPLC, and eluted at a flow rate of 4 mL/min with a linear gradient of 0–100% B, where solvent A is 100% H_2_O/0.1% trifluoroacetic acid (TFA) and solvent B is 100% acetonitrile/0.1%TFA. On HPLC, scuPA was eluted as a single peak under these conditions with a retention time of ~26 min. Yields were ranged from 10–20 mg protein per liter of S2 culture supernatant.

### Binding assays for suPAR ligands interaction

An anti-uPAR antibody (ATN658) was mixed with Protein A Dynabeads (Invitrogen) in PBST binding buffer (PBS plus 0.05% Tween20) at room temperature for 10 min. After being washed with the binding buffer, the Dynabeads were collected with a magnetic stand, and excess liquid was removed. The ATN658-coated beads were then mixed with monomeric or dimeric suPAR at room temperature for 10 min, followed by the washing of the beads. Different ligands, including ATF, SDH, vitronectin and HMWK, each at concentrations of 0.5 mg/mL, were then incubated with the beads for 10 min. After being washed three times with binding buffer, the bound protein complexes were eluted with an elution buffer (50 mM Glycine pH 2.8), and were analyzed on a 4–15% SDS precast polyacrylamide gel.

### Purification of suPAR:ATF complex

To obtain the dimeric or the monomer suPAR:ATF complex, suPAR dimer or monomer was mixed with excess ATF. After incubation at room temperature for 30 min, the mixture was purified on the gel filtration column (Superdex 200 10/300) with a buffer of 20 mM Tris pH 7.4, 150 mM KCl, at flow rates of 0.5 mL/minute.

### Surface plasmon resonance

Direct binding studies were carried out in real-time on a Biacore T200 instrument (Biacore, Uppsala, Sweden). In all experiments, 10 mM HEPES, 150 mM NaCl, 3 mM EDTA, and 0.005% (v/v) surfactant P-20 at pH 7.4 was used as running buffer. uPAR monoclonal antibody, ANT658, was immobilized on a CM5 sensor chip at two different levels of 10 and 20 μg/mL. Covalent coupling was performed with *N*-hydroxy-succinimide/*N*-ethyl-*N*-[3-(diethylamino) propyl]-carbodiimide in 10 mM sodium acetate, pH 4.5, at a flow rate of 5 μL/min for a target level 10000 RU. To obtain optimal preparations for binding kinetics valuation, both suPAR monomer and dimer were purified by size-exclusion chromatography (Superdex75) in Biacore running buffer prior to analysis to remove any traces of aggregated material. Soluble uPAR monomer or dimer (350 ng) was then captured onto the sensor chip for 3 min. Serial 3-fold dilutions of scuPA (0.54–44.4 nM)or ATF (0.14–33.3 nM) were subsequently passed over two flow cells with immobilized ANT658 captured with either suPAR monomer or dimer, and one mock coupled flow cell. Association was recorded for 120 s followed by a dissociation phase of 600 s at a flow rate of 30 μL/min at 20 °C. After each run, the sensor chip was regenerated by injection of Glycine2.0. The kinetic rate constants, K_on_ and K_off_, were derived from these real-time interaction analyses by fitting the association and dissociation phases to a simple bimolecular interaction model using the BIA evaluation software (Biacore).

### Crystallization and crystal structural determination

Crystals of dimeric suPAR were grown at 22 °C by sitting drop vapor diffusion with equal volumes of dimeric suPAR at 15 mg/mL and a precipitant solution of 1.8–2.2 M ammonium sulfate in 50 mM sodium acetate at pH 4.5–5.2. These crystals appear in rhombic shape. The crystals for data collection were flash-frozen in liquid nitrogen using a cryo-solution with the crystallization solution containing 25% glycerol. Data of the crystals were collected at a wavelength of 0.979 Å, corresponding to the “Se peak” absorption edge on beamline 22-ID at the Advanced Photon Source of Argonne National Laboratory, and integrated and scaled using HKL2000^61^. The structure was solved by single-wavelength anomalous dispersion (SAD) phasing implemented in CRANK^62^ pipeline within CCP4 program suite^63^. Selenium atom sites were identified using SHELXD^64^, and their positions were refined using BP3^65^. The correct hand for the phases was identified using SOLOMON^66^, and density modification was carried out in PARROT^67^ before atomic model building in BUCCANEER^68^. The partially refined model was iteratively improved by rounds of manual fitting using COOT^69^ and refinement using PHENIX refine^70^. In the final stages of refinement, TLS refinement was performed. The final model was refined at 2.91 Å to Rfactor/Rfree of 23.75%/28.97%. Details of the phasing and refinement statistics are shown in Table S4. In this structure, many residues, including 51–58, 79–152, 228–234 and 251–259, were missed in the model.

To obtain the crystals close to physiological pH, the crystals that grew under low pH were soaked in the buffer at pH 7.4 for 2 days, and flash-frozen with liquid nitrogen using a cryo-solution with the soaking solution contained 25% glycerol. Data of crystal were collected at a wavelength of 0.979 Å on beamline BL17U at Shanghai Synchrotron Radiation Facility. Diffraction datasets obtained from socked crystals were processed using the automated data processing pipeline Xia2^71^ with options that run XDS^72^. The structure was solved with MOLREP using the low pH structure as template. After refinement at 2.96 Å, the R-factor was 24.79% and the R-free 29.95%. The data obtained from the crystals soaked at neutral pH displayed more electron density, allowing to build in more residues into the model. The change of the pH-induced slight position movement of D1 N-terminal four β-strands (residue 1–49) (Supplementary Fig. 11), but the overall organization of uPAR dimer remained the same.

Statistics of the data collection, refinement and model validation are shown in Supplementary Table 4. The coordinates of the structures have been deposited in the Protein Data Bank (PDB) with the accession code 7V6 (acid pH) and 7E17 (neutral pH). Structural analyses were carried out using COOT^69^ and CCP4^63^, and graphic representations were prepared using PyMol (Schrödinger, Inc.).

### Plasmid construction

The pLVX-suPAR-mRuby3 plasmid was constructed by cloning the coding region from pcDNA3.1-suPAR-mRuby3-GPI into pLVX-IRES-Puro vector using restriction enzymes EcoRI and XhoI. The pLVX-Flag-uPAR was constructed by cloning the coding region from cDNA of A549 cells into pLVX-Flag-IRES-Puro vector using restriction enzymes EcoRI and XhoI, which was derived from pLVX-IRES-Puro vector. Site-directed mutagenesis was performed by PCR-based methods. Primers were designed with the desired mutations for introducing single-site mutation to the residues 47–49 of suPAR on the template pLVX-suPAR-mRuby3.

### Chemical cross-linking assays

Cross linking was performed by incubating 293T cells with 0.5 mM chemical cross-linking agent BS3 in PBS for 30 min at 4 °C. Un-reacted BS3 was removed by washing the cells with PBS, and the cells lysed as described below.

### Lentivirus packaging and stable cell line construction

Lentivirus was packaged by co-transfection (FuGENE from Promega) into HEK293T cells with plasmids of pLVX-suPAR-mRuby3, its mutants or pLVX-mRuby3, VSV-G (Addgene, #8454), PRRE (Addgene, #12251) and REV (Addgene, #12253). The transfected medium was changed into fresh DMEM after 8–12 h, and lentivirus-containing supernatants were collected 48 h later, followed by passing through a 0.45-µm filter and diluted 1:1 with fresh medium containing 8 µg/mL polybrene, and were then used directly to infect the target cells (HEK293T) at 70~80% confluence. Selection antibiotic puromycin (Invitrogen) was added at its killing concentration of 1–2 μg/mL. Fresh media with antibiotics was added every two days until all the cells were dead in the killing control.

### Immunoprecipitation

293T cells were transfected for 36 h with the appropriate plasmids and were lysed in ice-cold lysis buffer (5% glycerol, 50 mM Tris-HCl pH 7.4, 150 mM NaCl, and 1% Triton X-100, containing 10 mM NaF, 2 mM Na_3_VO_4_, 10 µg/mL leupeptin and 1 µM PMSF). The cell lysates were subjected to immunoprecipitation with anti-Flag beads (Anti-DYKDDDDK Affinity Beads, Smart Life Sciences), and were eluted by boiling in 1X SDS loading buffer for 10 min. The proteins were detected by immunoblotting analysis with the appropriate antibodies (identified below).

### Immunoblotting analysis

Cells were lysed directly in 2X SDS loading buffer (diluting from 4X SDS loading buffer containing 250 mM Tris-HCl (pH 6.8), 8% SDS, 0.2% bromophenol blue, 40% glycerol and 20% β-mercaptoethanol with cell lysis buffer as shown above), and boiled for 10 min. Proteins were separated by SDS-PAGE, transferred onto a nitrocellulose membrane and then identified by immunoblotting analysis with the appropriate primary antibodies as stated below. Anti-GAPDH (1:3000 dilution), anti-phospho-ERK1/2 (1:1000 dilution), anti-ERK1/2 (1:10,000 dilution), polyclonal anti-uPAR (1:500 dilution) and horseradish peroxidase-conjugated antibody to rabbit IgG or to mouse IgG (1:5000 dilution for each) were used in each blot. The protein bands were visualized with a Meilunbio® fg super sensitive ECL luminescence reagent (MA0186) according to the manufacturer’s instructions.

### Cell proliferation assay using CCK-8

The cell count was measured by Cell Counting Kit-8 (CCK-8) (Meilunbio). Briefly, 500 cells were seeded in a 96-well plate and cultured till 5 days. After incubation in CO_2_ incubator for 24 h, 10 μl of CCK-8 solution was added to each well, and the 96-well plate was further incubated at 37 °C for 1 h. Normal DMEM with CCK-8 was served as the blank control. Meanwhile, the standard curve for cell count was generated every day. The cell count was calculated according to the standard line by measuring OD_450_ on a microplate reader (SpectraMax® i3x, Molecular Devices). All doses were carried out in triplicates.

### High content imaging analysis

For the ATF-binding assay, cells were pre-treated with ATF-FITC for 1 h or 6 h and incubated with Hoechst 33342 for 10 min before fixation and imaging on the Operetta High Content Imaging System (Perkin Elmer). Images from sixteen fields per well were taken with a ×63/NA1.15 objective using the laser confocal method on three channels with these settings for each fluorescent molecule: Hoechst 33342 (exposure: 20 ms, excitation: 350 nm/50 nm, emission: 455 nm/50 nm), FITC (exposure: 1 s, excitation: 490 nm/20 nm, emission: 525 nm/36 nm) and mRuby3 (exposure: 1 s excitation: 543 nm/22 nm, emission: 605 nm/64 nm). Cell images were then analyzed by the Operetta CLS software using the multi-target analysis. First, cells were identified using the nuclear dye channel (minimum area 70 μm and sensitivity of 40), and their cytoplasm was defined by either the mRuby3 channel. Cell counts were measured from the nuclear dye. Next, the FITC intensity in the cytoplasmic region was calculated, and divided by the cytoplasmic mRuby3 intensity, yielding the ATF-binding ratio.

For the uPAR distribution assay, cells were incubated with ATF for 6 h, followed by incubation with Hoechst 33342 for 10 min before fixation. Cell imaging was performed on the Operetta High Content Imaging System (Perkin Elmer). Sixteen fields per well were taken with a ×63/NA1.15 objective using the laser confocal method on two channels with these settings for each fluorescent molecule: Hoechst 33342 (exposure: 20 ms, excitation: 350 nm/50 nm, emission: 455 nm/50 nm) and mRuby3 (exposure: 1 s, excitation: 543 nm/22 nm, emission: 605 nm/64 nm). The images of 20 layers on the mRuby3 channel for 10 μm total vertical distance from the basal were recorded. Cell images were then analyzed by the 3D analysis module of Operetta CLS software. Object identification was performed using the nuclear dye images (minimum area 70 μm and sensitivity of 40). Cell counts were measured from the nuclear dye. The experimental parameters measured from the mRuby3 images included cytoplasmic mRuby3 intensity. The uPAR distribution ratio was measured by the mRuby3 intensity of apical layers (15 layers) and basal layers (5 layers) compared to the mRuby3 intensity of the whole cell. The movies were built by combining images of the mRuby3 (Red) and Hoechst (Blue) using 3D analysis module.

For the cytoskeleton assay, cells were cultured in CellCarrier-96-well plate (PerkinElmer) 24 h and fixed with 3.7% paraformaldehyde for 10 min, washed with PBS for 5 min three times, and permeabilized with 1% Triton X-100 in PBS, followed by blocking with a solution containing 0.1% Triton X-100 and 5% BSA in PBS. F-actin was stained with Actin-Tracker Green (FITC-labelled phalloidin, Beyotime, China) in the blocking buffer. After incubation for 1 h at room temperature, the cells were washed with PBS for 5 min three times, and imaged on the Operetta High Content Imaging System. Sixteen fields per well were taken with a ×63/NA1.15 objective using the laser confocal method on three channels with these settings for each fluorescent molecule: Hoechst 33342 (exposure: 20 ms, excitation: 350 nm/50 nm, emission: 455 nm/50 nm), FITC (exposure: 500 ms, excitation: 490 nm/20 nm, emission: 525 nm/36 nm), and mRuby3 (exposure: 1 s, excitation: 543 nm/22 nm, emission: 605 nm/64 nm).

For the cell proliferation assay, live-cell imaging was performed on the Operetta High Content Imaging System (Perkin Elmer). Sixteen or tween five fields per well were taken with a ×20/0.45 water objective using the noncofocal method on Digital Phase Contrast channel. Images were recorded every half an hour for 3 days in the cell culture condition (37°C, 5% CO_2_) on Perkin Elmer high content imager. Cell images were then analyzed by the Operetta CLS software using the Cell Division analysis. Cell proliferation curve was generated with cell counts by analyzing images of DPC for different cell lines.

### Quantification and statistical analysis

All data are representative of at least three independent experiments. The data are presented as the mean ± SD as indicated in the legends. The two-tailed unpaired Student’s *t* test was used to compare the ATF-binding ratio between groups in ATF-binding assays. A *p*-value < 0.05 was considered statistically significant.

### Reporting summary

Further information on research design is available in the Nature Research Reporting Summary linked to this article.

## Supplementary information

Supplementary Information

Description of Additional Supplementary Files

Supplementary Movie 1

Supplementary Movie 2

Supplementary Movie 3

Supplementary Movie 4

Supplementary Movie 5

Supplementary Movie 6

Reporting Summary

## Data Availability

All data generated and analyzed in this study are included in the Article and its Supplementary Information, and are also available from the corresponding authors upon reasonable request. The X-ray crystallographic coordinates for structures reported in this study have been deposited in the Protein Data Bank (PDB) under accession codes 7E17 (Structure of dimeric uPAR), 7V63 (Structure of dimeric uPAR at low pH) and 3BT2 (Structure of ligand-bound monomeric uPAR). Source data are provided with this paper.

## Source data

Source-data
