# Supplementary material for: Crystal structure and cellular functions of uPAR dimer
Source: Nat Commun. 2022 Mar 29;13:1665. doi: 10.1038/s41467-022-29344-y (PMC8964761; doi:10.1038/s41467-022-29344-y)
Supplement: Supplementary file 1 — Supplementary Information [file 41467_2022_29344_MOESM1_ESM.pdf]

Supplementary Information
for
Crystal Structure and Cellular Functions of uPAR Dimer

Shujuan Yu[1,2], Yaqun Sui[1], Jiawei Wang[3], Yongdong Li[1], Hanlin Li[1], Yingping Cao[4], Liqing Chen[5], Longguang Jiang[1], Cai Yuan[2,6*], Mingdong Huang[1,2*]

[1]College of Chemistry, Fuzhou University, Fuzhou, Fujian, 350116, China

[2]College of Biological Science and Engineering, Fuzhou University, Fuzhou, Fujian, 350116, China

[3]Tsinghua University, Beijing, 100084, China;

[4]Department of Clinical Laboratory, Fujian Medical University Union Hospital, Fujian, China

[5]School of Molecular Sciences, Arizona State University, Tempe, Arizona, 85287, United States

[6]Key Laboratory of Marine Enzyme Engineering, Fuzhou University, Fuzhou, Fujian 350116, China

[*] Corresponding authors: Yuan C. (cyuan@fzu.edu.cn) and Huang M. (hmd_lab@fzu.edu.cn)

Supplementary Table 1. Hydrogen bonds between two protomers in suPAR dimer

Number	Chain A contact atom	Location in chain A	Chain B contact atom	Location in chain B	Distance, Å
1	R2 O	β1A	L75 N	β1FG loop	2.67
2	R2 NE	β1A	D74 OD1	β1FG loop	3.62
3	R2 N	β1A	D74 OD1	β1FG loop	2.53
4	M4 O	β1A	N77 ND2	β1FG loop	2.97
5	M4 N	β1A	N77 OD1	β1FG loop	2.89
6	C24 SG	β1C	L73 O	β1FG loop	3.79
7	C24 O	β1C	C71 N	β1FG loop	3.35
8	C24 N	β1C	C71 O	β1FG loop	2.72
9	R25 O	β1C	N77 ND2	β1FG loop	3.01
10	R25 NH1	β1C	E183 OE1	D1D2 loop	3.69
11	R25 NH1	β1C	E185 OE1	D1D2 loop	3.45
12	T26 OG1	β1C	N77 O	β1FG loop	3.80
13	T26 O	β1C	V69 N	β1F	2.84
14	T26 N	β1C	V69 O	β1F	2.84
15	I28 O	β1C	T67 N	β1F	2.78
16	I28 N	β1C	T67 O	β1F	2.62
17	R30 O	β1C	S65 N	β1F	2.93
18	R30 N	β1C	S65 O	β1F	2.91
19	W32 O	β1C	I63 N	β1F	2.75
20	W32 N	β1C	I63 O	β1F	2.87
21	G35 O	β1CD loop	L93 N	β1G	2.86
22	E36 OE2	β1D	S90 OG	β1G	3.52
23	E37 O	β1D	R91 N	β1G	2.94
24	E37 N	β1D	R91 O	β1G	2.83
25	E39 O	β1D	R89 N	β1G	2.90
26	E39 N	β1D	R89 O	β1G	2.89
27	V41 N	β1D	Y87 O	β1FG loop	2.61
28	S44 O	β1D	N77 ND2	β1FG loop	3.55
29	T46 OG1	β1D	E185 OE1	D1D2 loop	3.43
30	T51 OG1	β1DE loop	N186 ND2	D1D2 loop	3.40
31	T51 O	β1DE loop	N186 ND2	D1D2 loop	2.52
32	T51 N	β1DE loop	E185 O	D1D2 loop	2.90
33	R53 NH1	β1E	N186 OD1	D1D2 loop	3.89
34	R58 NH1	β1EF loop	LYS175 O	α2G	3.39
35	T59 N	β1EF loop	E178 OE1	α2G	2.58
36	G60 O	β1EF loop	T174 OG1	α2G	2.35
37	G60 N	β1EF loop	E178 OE1	α2G	2.29
38	I63 O	β1F	W32 N	β1C	3.16
39	I63 N	β1F	W32 O	β1C	3.13
40	S65 O	β1F	R30 N	β1C	3.06
41	S65 N	β1F	R30 O	β1C	3.02

42	T67 O	β1F	I28 N	β1C	2.78
43	T67 N	β1F	I28 O	β1C	2.94
44	E68 OE2	β1F	R25 NH1	β1C	3.46
45	E68 OE2	β1F	T27 OG1	β1C	3.73
46	V69 O	β1F	T26 N	β1C	2.99
47	V69 N	β1F	T26 O	β1C	3.04
48	C71 SG	β1F	C24 O	β1C	3.87
49	C71 O	β1F	C24 N	β1C	2.94
50	C71 N	β1F	C24 O	β1C	3.25
51	L73 O	β1FG loop	C24 SG	β1C	3.77
52	D74 OD2	β1FG loop	R2 NH1	β1A	3.55
53	D74 OD1	β1FG loop	R2 N	β1A	2.69
54	L75 N	β1FG loop	R2 O	β1A	2.78
55	N77 OD1	β1FG loop	M4 N	β1A	2.77
56	N77 ND2	β1FG loop	M4 O	β1A	2.86
57	N77 ND2	β1FG loop	R25 O	β1C	2.95
58	Y87 O	β1FG loop	V41 N	β1D	2.78
59	S88 OG	β1G	T8 OG1	β1AB loop	2.79
60	S88 N	β1G	T8 OG1	β1AB loop	3.67
61	R89 O	β1G	E39 N	β1D	3.04
62	R89 N	β1G	E39 O	β1D	2.98
63	S90 OG	β1G	E36 OE2	β1D	3.04
64	R91 O	β1G	E37 N	β1D	2.72
65	R91 N	β1G	E37 O	β1D	2.84
66	L93 N	β1G	G35 O	β1D	2.90
67	T174 OG1	α2G	G60 O	β1EF loop	2.53
68	E178 OE2	α2G	L61 N	β1EF loop	3.88
69	E178 OE1	α2G	T59 N	β1EF loop	2.97
70	E183 OE1	D1D2 loop	R25 NH1	β1C	3.81
71	E185 OE1	D1D2 loop	R25 NH1	β1C	3.05
72	E185 O	D1D2 loop	T51 OG1	β1DE loop	3.63
73	E185 O	D1D2 loop	T51 N	β1DE loop	3.14
74	N186 ND2	D1D2 loop	T51 O	β1DE loop	2.55
75	N186 ND2	D1D2 loop	T51 OG1	β1DE loop	3.35
76	R216 NH2	β3BC loop	S48 O	β1DE loop	2.71

Supplementary Table 2. Salt bridges between two protomers in suPAR dimer

Number	Chain A contact atom	Location in chain A	Chain B contact atom	Location in chain B	Distance, Å
1	R2 NE	β1A	D74 OD1	β1FG loop	3.62
2	R2 NE	β1A	D74 OD2	β1FG loop	3.85
3	R25 NH1	β1C	E183 OE1	D1D2 loop	3.69
4	R25 NH1	β1C	E185 OE1	D1D2 loop	3.45
5	K62 NZ	β1F	E33 OE1	β1C	3.97
6	E68 OE2	β1F	R25 NH1	β1C	3.46
7	D74 OD1	β1FG loop	R2 NH1	β1A	3.86
8	D74 OD2	β1FG loop	R2 NH1	β1A	3.55
9	E183 OE1	D1D2 loop	R25 NH1	β1C	3.81
10	E185 OE1	D1D2 loop	R25 NH1	β1C	3.05
11	E185 OE2	D1D2 loop	R25 NH1	β1C	3.94

Supplementary Table 3. List of oligonucleotide primers

Primer name	DNA sequence[a]
Plaur full-length (uPAR)-f	GACGATAAG**GAATTC**ATGGGTCACCCGCCGCTG
Plaur full-length (uPAR)-r	GAACTAGT**CTCGAG**TTAGGTCCAGAGGAG
uPAR-f	TTTCCGGT**GAATTC**ATGAAGTGGGTAACCTTT
uPAR-r	GAACTAGT**CTCGAG**TTAGGTCCAGAGGAG
uPAR H47A-f	AGAAAAGCTGTACC**GC**CTCAGA
uPAR H47A-r	GTTGGTCTTCTCTGAG**GC**GGTAC
uPAR H47C-f	AGCTGTACCTGCTCA**GAG**AAGACCAACAGG
uPAR H47C-r	CTT**CTC**TGAGCAGGTACAGCTTTTCTCCAC
uPAR H47F-f	AGAAAAGCTGTACC**TT**CTCAGA
uPAR H47F-r	GTTGGTCTTCTCTGAG**AA**GGTAC
uPAR S48P-f	AGCTGTACCCAC**C**CAGAGA
uPAR S48P-r	TGTTGGTCTTCTCTG**G**GTGG
uPAR E49A-f	AGCTGTACCCACTCA**GCG**AAGAC
uPAR E49A-r	GTCCTGTTGGTCTT**CGC**TGAGT
uPAR E49F-f	GCTGTACCCACTCA**TTC**AAGAC
uPAR E49F-r	GTCCTGTTGGTCTT**GAA**TGAGTG
uPAR E49P-f	CACTCA**CC**GAAGACCAACAGGACCCTGA
uPAR E49P-r	GTCTTC**GG**TGAGTGGGTACAGCTTTTCT
uPAR T51C-f	GAGAAG**TG**CAACAGGACCCTGAGCTATCG
uPAR T51C-r	CTGTTG**CA**CTTCTCTGAGTGGGTACAGCT
uPAR V70C-f	GAGGTT**TGT**TGTGGGTTAGACTTGTGCAA
uPAR V70C-r	ACCCACA**ACA**AACCTCGGTAAGGCTGGTGAT
uPAR N259C-f	TTCAGCATG**TG**CCACATTGATGTCTCCTGC
uPAR N259C-r	ATCAATGTGG**CA**CATGCTGAAGGCGTCACC

[a]The restriction sites and the mutant sites were underlined and in bold.

Supplementary Table 4. Data collection and refinement statistics of dimeric suPAR crystals at two pH values.

Crystals	pH 4.6	pH 7.4
**Data collection and scaling**		
Beamline	NSBL X29	SSRF BL17U
Spacegroup	$P4_322$	$P4_32_12$
	a = 77.45 Å	a = 79.58 Å,
Cell parameters	b = 77.45 Å	b =79.58 Å,
	c =275.53 Å	c = 270.53 Å
Wavelength (Å)	0.979	0.979
Resolution (Å)	47.04- 2.91 (3.01- 2.91)	55.09 - 2.96 (3.066 - 2.96)
Rsym or Rmerge	0.126 (0.313)	0.175 (1.23)
Number of unique reflections	17341 (839)	18913 (1347)
$I/\sigma(I)$	9.87 (3.24)	10.09 (2.16)
Completeness (%)	89.23 (44.58)	99.25 (99.95)
Multiplicity	9.0 (5.3)	6.7(7.3)
**Phasing**		
Number of sites	12	
figure of merit	0.384	
**Refinement**		
R_{work}	0.2375 (0.2512)	0.2479 (0.3259)
R_{free}	0.2897 (0.3937)	0.2995 (0.3345)
Average B factor (Å^2)	81.50	61.34
Protein residues	376	457
**Validation**		
RMS deviations from ideal		
Bond lengths (Å)	0.010	0.010
Bond angles (°)	1.59	1.26
Ramachandran plot		
Ramachandran favored (%)	91	91.3
Ramachandran outliers (%)	1.7	0

Values in parentheses are given for the highest resolution shells.

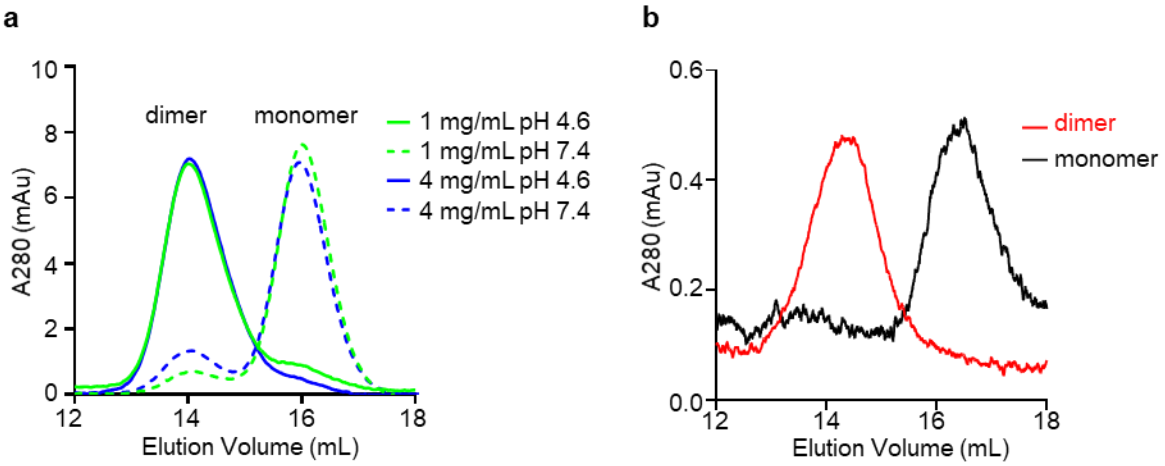

Supplementary Figure 1. Recombinant suPAR formed dimer at high concentration and low pH, and dimeric suPAR was stable in low concentration at neutral condition. (a) Gel filtration detected the formation of suPAR dimer at 1mg/mL and 4 mg/mL under different pH. suPAR was dissolved to 1 mg/mL (green) and 4 mg/mL (blue) at low pH (pH 4.6, solid lines) or neutral pH (pH 7.4 dash lines) for 1 day, and was analyzed on a gel filtration column (superdex200). (b) suPAR dimer was stable in low concentration at neutral condition. Recombinant suPAR dimer or monomer was diluted into low concentration (3.5 µg/mL) under neutral pH (pH 7.4) in the buffer containing 20 mM Tris-HCl pH 7.4, 150 mM NaCl for 1 day at RT and was analyzed on a gel filtration column (superdex200). Source data are provided as a Source Data file.

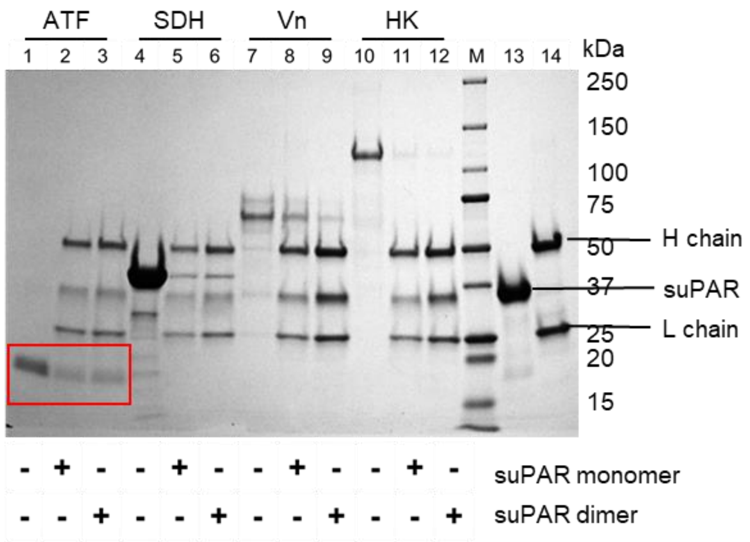

Supplementary Figure 2. Either suPAR monomer or dimer bound to the ligands, ATF, SDH (streptococcal surface dehydrogenase), Vn (vitronectin), and HK (high molecular weight kininogen). The monomer or dimer was loaded onto ATN658 bound Protein A Dynabeads. Different ligands were then bound and eluted with a low pH buffer after wash. The bands corresponding to ATF were highlighted in the red box. The eluents were analyzed by a 4-15% SDS–PAGE. Source data are provided as a Source Data file.

a

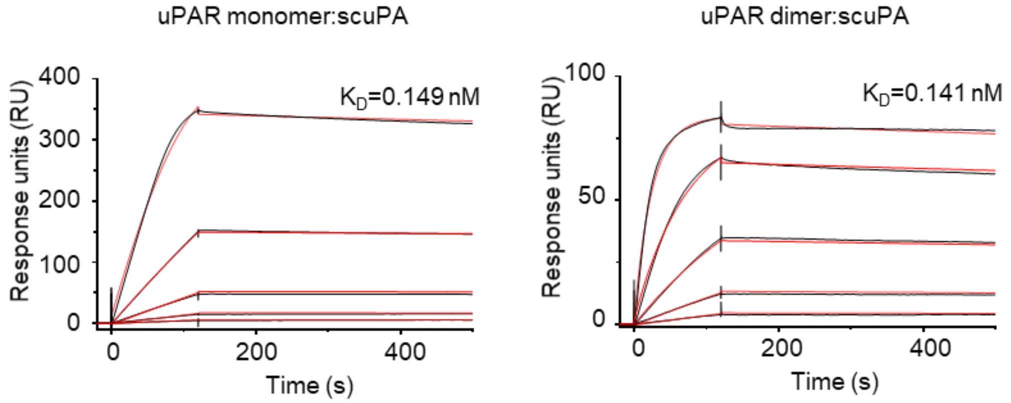

b

	K_{on} (1/Ms)	K_{off} (1/s)	K_D (10^{-9} M)
uPAR monomer:scuPA	770263.3	0.000115	0.149
uPAR dimer:scuPA	917423.2	0.000129	0.141

Supplementary Figure 3. Either suPAR monomer or dimer bound to scuPA with similar high affinity. (a) Kinetics of the ligand:uPAR (either suPAR monomer or suPAR dimer) interaction by surface plasmon resonance. Serial 3-fold dilutions of purified recombinant scuPA were allowed to interact with immobilized ANT658 captured suPAR monomer or suPAR dimer at 25 °C and a flow rate of 30 μL/min. Sensorgrams recorded for the association (120 s) and dissociation (600 s) of the various scuPA concentrations are shown as solid black lines with the corresponding curves fitting to a 1:1 Langmuir binding superimposed as solid red lines. The derived kinetic rate constants are shown in (b). Source data are provided as a Source Data file.

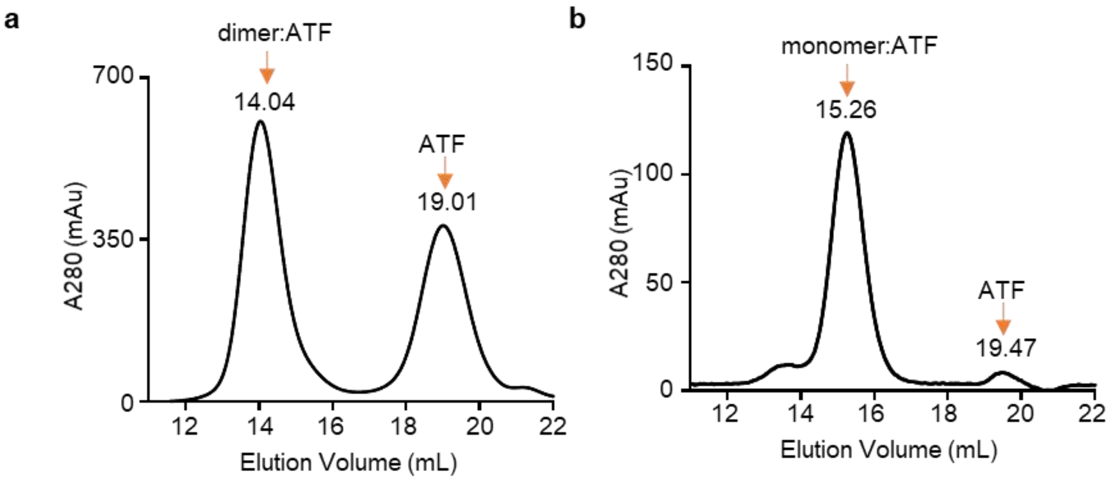

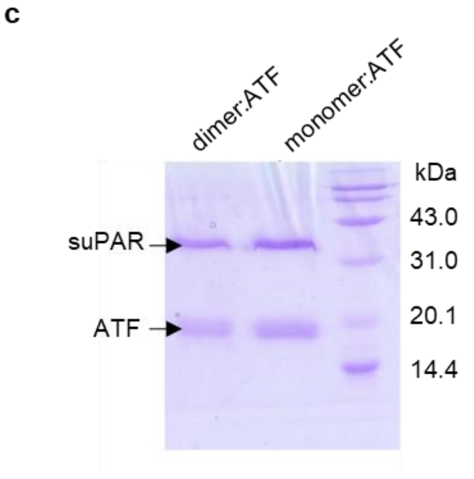

d

	dimer:ATF	monomer:ATF
suPAR band	11034.702	16994.97
ATF band	10407.43	15278.82
Ratio	1.060271556	1.112322

Supplementary Figure 4. ATF bound to dimeric suPAR at 2:1 molar ratio. Dimer:ATF complex (a) and monomer:ATF complex (b) were purified by gel filtration and identified by electrophoretic analysis with 15% SDS-PAGE (c). (d) The molar ratio of dimeric suPAR :ATF was analyzed with the ChemDoc Touch imaging system (Bio-Rad) using monomeric suPAR:ATF as the standard. Source data are provided as a Source Data file.

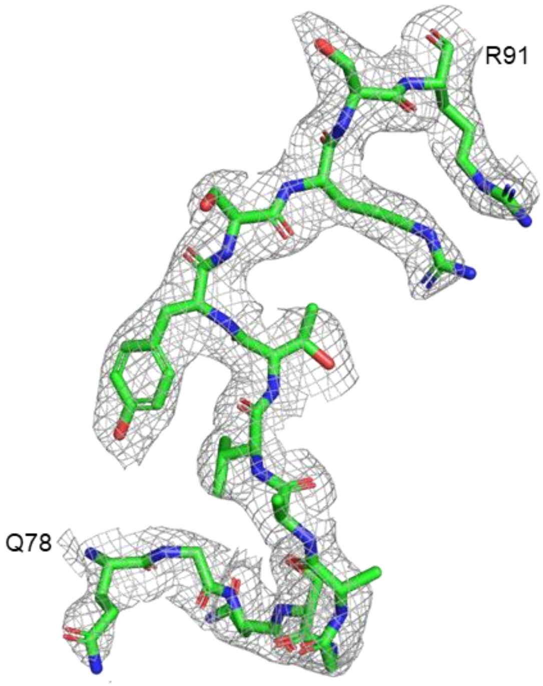

Supplementary Figure 5. Resi 78-91 with good electron density. 2Fo-Fc electron density map contoured at 1 sigma.

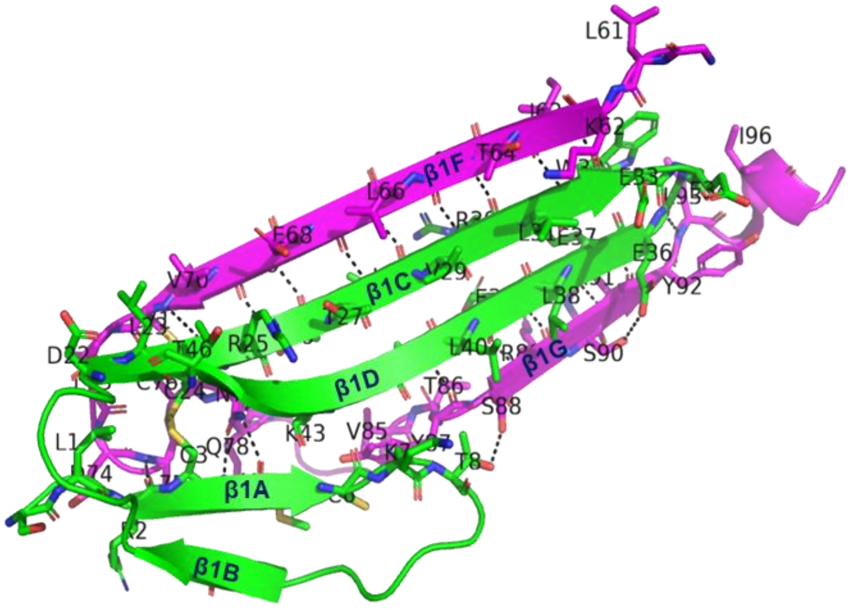

Supplementary Figure 6. The tight interaction of swapping domain 1 in suPAR dimer. Each protomer is colored as green and magenta, respectively.

a b

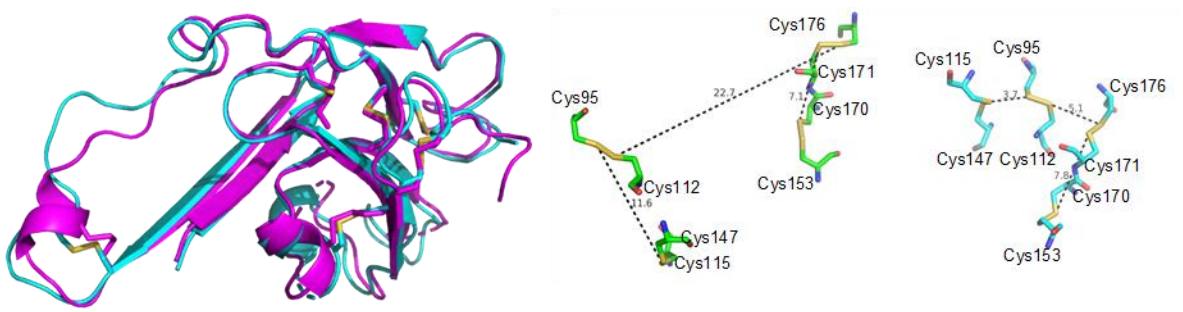

Supplementary Figure 7. Structure comparison of uPAR dimer and monomer. (a) Residues 155-277 of uPAR dimer (purple) and monomer (blue) adopt similar conformation. (b) The large repositioning of the disulfide bonds in the palm region of the D2 of dimeric suPAR (left) is seen compared to the highly conserved disulfide bonding pattern of the three-fingered fold (right)**.** The interaction residues between two molecules are shown as stick.

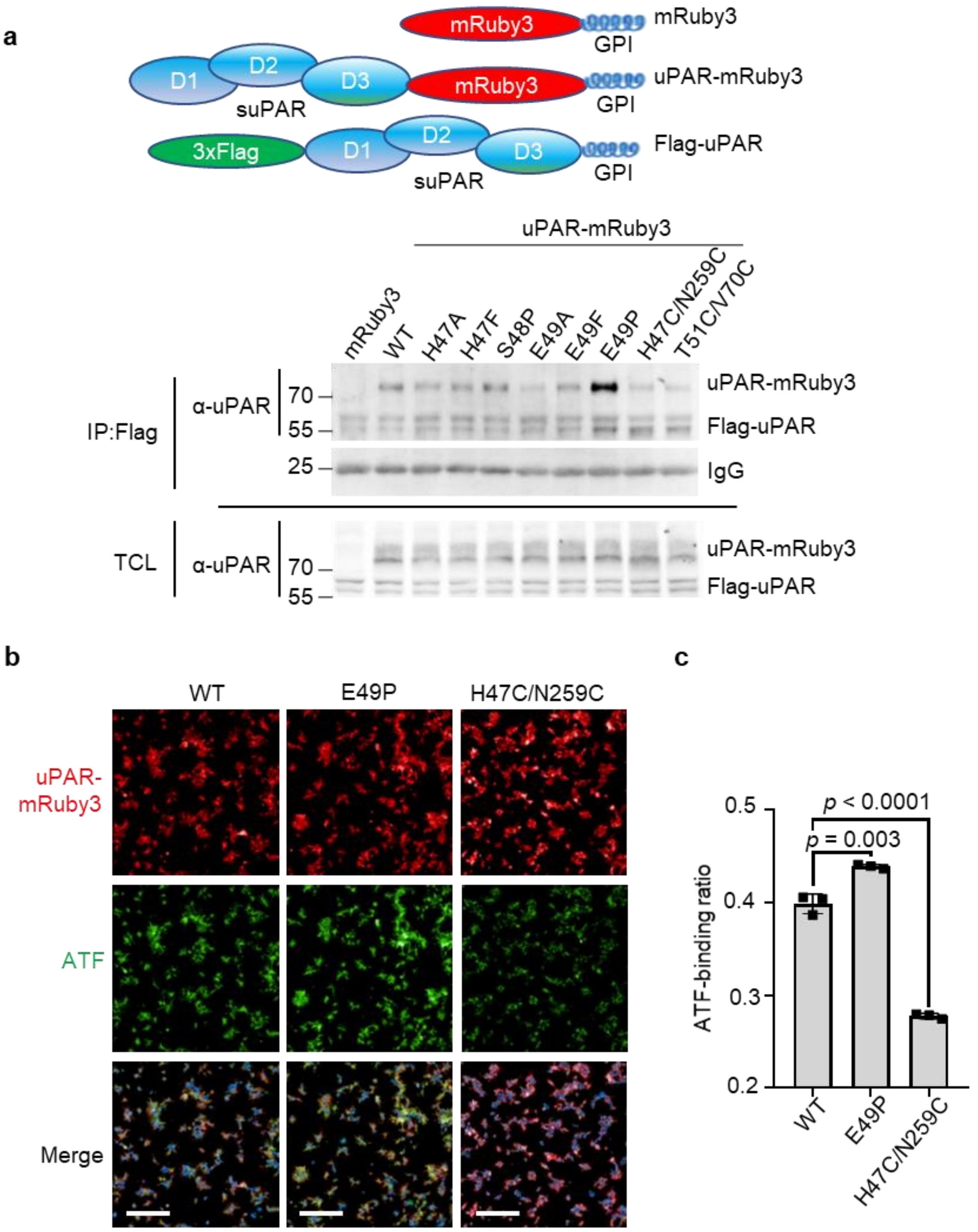

Supplementary Figure 8. The effect of hinge region mutation for uPAR dimerization. (a) Anti-uPAR immunoblotting of Flag-tagged uPAR and mRuby3-tagged uPAR mutants (top panel) expressed on 293T cells in the anti-Flag immunoprecipitants (IP: Flag) (above) and the total cell lysates (bottom) of 293T cells co-expressing Flag-tagged uPAR and mRuby3-tagged uPAR. (b) Microscopic images of the uptake of FITC-ATF by the uPAR-mRuby3 293T cells for 6 hr. Scale bars, 200 µm. (c) Quantification of ATF-binding ratio for b, which was calculated by Operetta CLS software. Data were representative of three independent experiments. Data are presented as mean +/− SD and the *p-values* of two-tailed unpaired Student's t test are indicated. Source data are provided as a Source Data file.

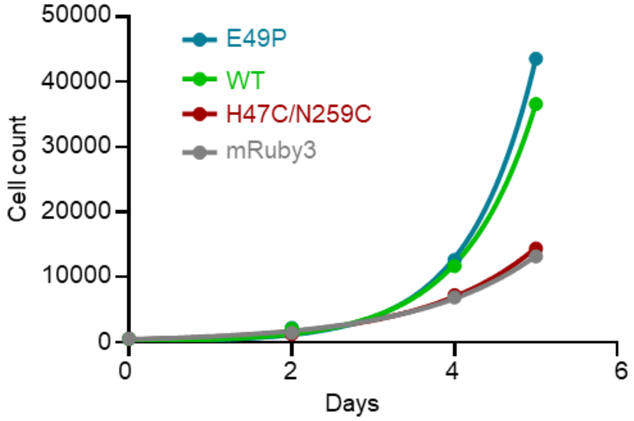

Supplementary Figure 9. Dimerization of uPAR on cell surface promoted cell proliferation. Cell proliferation of mRuby3-tagged uPAR 293T stable cell lines was measured with CCK-8 assay. Data are presented as mean +/− SD. Source data are provided as a Source Data file.

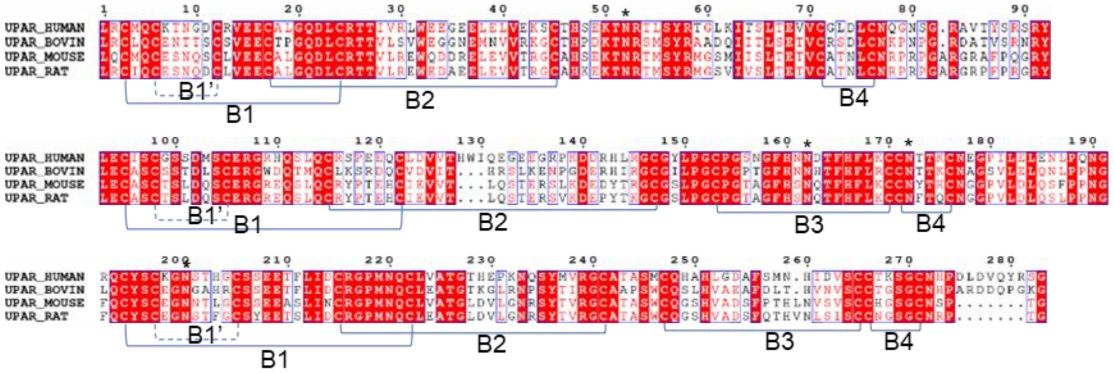

Supplementary Figure 10. Sequence alignments of the uPAR from different species. The consensus disulfide bonds in the palm of the three-fingered fold domain are shown in solid line, and disulfide bonds in the fingers of the three-fingered fold domain are shown in the dotted line. The potential glycosylation sites (N52, N162, N172 and N200) are marked with stars, which are conserved in these species and do not interfere with the dimer formation.

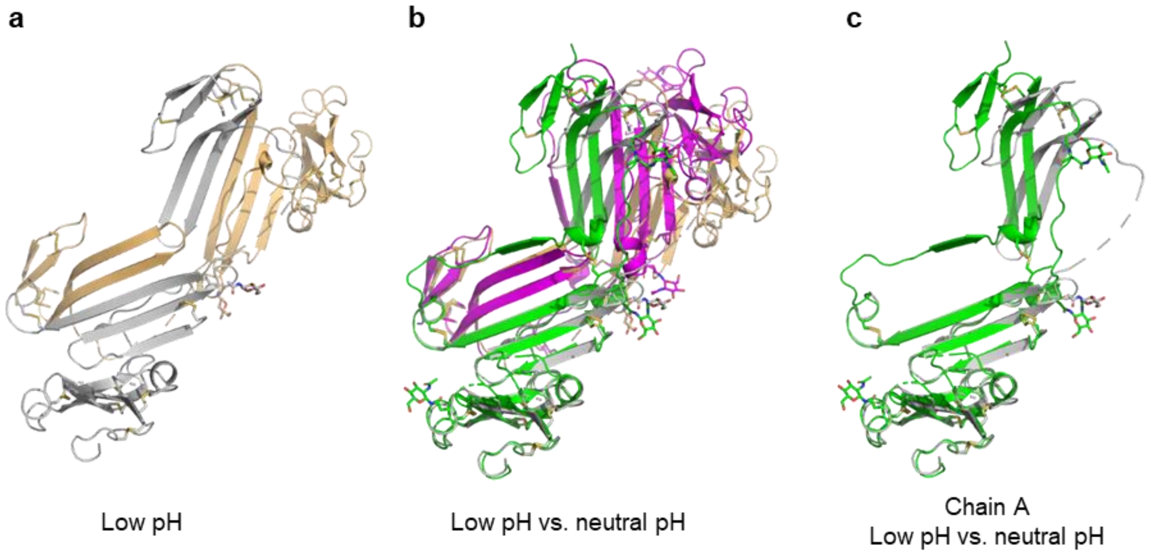

Supplementary Figure 11. Structure comparison of suPAR dimer at low pH and neutral pH. (a) The structure of suPAR dimer at low pH. The two protomers are colored grey and pale yellow. (b) Structure comparison of suPAR dimer at low pH and neutral pH. (c) Only chain A in b is displayed for simplicity.
