# Supplementary material for: Crystal structure and cellular functions of uPAR dimer
Source: Nat Commun. 2022 Mar 29;13:1665. doi: 10.1038/s41467-022-29344-y (PMC8964761; doi:10.1038/s41467-022-29344-y)
Supplement: Supplementary file 2 — Description of Additional Supplementary Files [file 41467_2022_29344_MOESM2_ESM.docx]

**Description of Additional Supplementary Files**

File Name: Supplementary Movie 1
Description: A movie of representative field showing three-dimensional distribution of uPAR (in red) on uPAR^WT^ 293T cells under normal culture condition, demonstrating that uPAR was asymmetrically distributed to basal areas of cells, nearby the bottom of culture plate. The movie was re-built by combining images of the uPAR (Red) and Hoechst (Blue) using 3D analysis module of Operetta CLS software. Z axis points from the basal side to the apical side of the cells.

File Name: Supplementary Movie 2
Description: E49P mutation caused uPAR (shown in red) to concentrate more onto the basal side (Z axis close to 0) of the uPAR^E49P^ cells compared to wide type uPAR. The nuclei of cells were stained in blue. The whitening area showed the corresponding uPAR level was higher than the red area. The quantitation of this conclusion was shown in Figure 4. The movie was re-built by the 3D analysis module of Operetta CLS software.

File Name: Supplementary Movie 3
Description: Relatively uniform distribution of uPAR in three dimensions on uPAR^H47C/N259C^ 293T cells under normal culture condition. The nuclei of cells were stained in blue. The mutations of H47C/N259C restrict uPAR in a monomeric form. The movie was re-built with one representative field by the 3D analysis module of Operetta CLS software.

File Name: Supplementary Movie 4
Description: The addition of ATF promoted uPAR to re-distribute to basal layers of uPAR^WT^ 293T cells as shown by the representative movie field. The results of the average over 16 fields of cells were presented in Figure 4. The nuclei of cells were stained in blue. The whitening area showed the corresponding uPAR level was higher than the red area. The movie was re-built with one representative field by the 3D analysis module of Operetta CLS software.

File Name: Supplementary Movie 5
Description: uPAR^E49P^ moved to the basal layers of the uPAR^E49P^ 293T cells in the presence of ATF. The nuclei of cells were stained in blue. The whitening area showed the corresponding uPAR level was higher than the red area. The quantitation of this conclusion was shown in Figure 4. The movie was re-built with one representative field by the 3D analysis module of Operetta CLS software.

File Name: Supplementary Movie 6
Description: The three-dimensional distribution of uPAR monomer (in red) on uPAR^H47C/N259C^ 293T cells in the presence of ATF. The mutations of H47C/N259C restrict uPAR in a monomeric form. The nuclei of cells were stained in blue. The movie was re-built with one representative field by the 3D analysis module of Operetta CLS software.
