# Supplementary material for: Crystal structure and cellular functions of uPAR dimer
Source: Nat Commun. 2022 Mar 29;13:1665. doi: 10.1038/s41467-022-29344-y (PMC8964761; doi:10.1038/s41467-022-29344-y)
Supplement: Supplementary file 9 — Reporting Summary [file 41467_2022_29344_MOESM9_ESM.pdf]

nature research

Corresponding author(s): Cai Yuan and Mingdong Huang

Last updated by author(s): Dec 16, 2021

Reporting Summary

Nature Research wishes to improve the reproducibility of the work that we publish. This form provides structure for consistency and transparency in reporting. For further information on Nature Research policies, see our Editorial Policies and the Editorial Policy Checklist.

Statistics

For all statistical analyses, confirm that the following items are present in the figure legend, table legend, main text, or Methods section.

n/a	Confirmed	
☐	☒	The exact sample size (*n*) for each experimental group/condition, given as a discrete number and unit of measurement
☐	☒	A statement on whether measurements were taken from distinct samples or whether the same sample was measured repeatedly
☐	☒	The statistical test(s) used AND whether they are one- or two-sided<br>*Only common tests should be described solely by name; describe more complex techniques in the Methods section.*
☒	☐	A description of all covariates tested
☒	☐	A description of any assumptions or corrections, such as tests of normality and adjustment for multiple comparisons
☐	☒	A full description of the statistical parameters including central tendency (e.g. means) or other basic estimates (e.g. regression coefficient) AND variation (e.g. standard deviation) or associated estimates of uncertainty (e.g. confidence intervals)
☐	☒	For null hypothesis testing, the test statistic (e.g. *F*, *t*, *r*) with confidence intervals, effect sizes, degrees of freedom and *P* value noted<br>*Give P values as exact values whenever suitable.*
☒	☐	For Bayesian analysis, information on the choice of priors and Markov chain Monte Carlo settings
☒	☐	For hierarchical and complex designs, identification of the appropriate level for tests and full reporting of outcomes
☒	☐	Estimates of effect sizes (e.g. Cohen's *d*, Pearson's *r*), indicating how they were calculated

Our web collection on statistics for biologists contains articles on many of the points above.

Software and code

Policy information about availability of computer code

Data collection	ÄKTA™pure; Biacore T200 instrument (Biacore, Uppsala, Sweden); ChemDoc Touch imaging system (Bio-Rad); Operetta High Content Imaging System (Perkin Elmer).
Data analysis	UNICORN7.0; Biacore Evaluation Software 3.1; Image lab 5.2(Bio-Rad); HKL2000; XIA2 0.3.5.0; CCP4 7.1; PHENIX 1.19_4092; Coot 0.8.9.2; PyMOL1.3; Operetta CLS software(Harmony 4.9).

For manuscripts utilizing custom algorithms or software that are central to the research but not yet described in published literature, software must be made available to editors and reviewers. We strongly encourage code deposition in a community repository (e.g. GitHub). See the Nature Research guidelines for submitting code & software for further information.

Data

Policy information about availability of data

All manuscripts must include a data availability statement. This statement should provide the following information, where applicable:

- Accession codes, unique identifiers, or web links for publicly available datasets
- A list of figures that have associated raw data
- A description of any restrictions on data availability

All data generated and analyzed in this study are included in the Article and its Supplementary Information, and are also available from the corresponding authors upon reasonable request. The X-ray crystallographic coordinates for structures reported in this study have been deposited in the Protein Data Bank (PDB) under accession codes 7E17 (Structure of dimeric uPAR, DOI: 10.2210/pdb7e17/pdb) and 7V63 (Structure of dimeric uPAR at low pH, DOI: 10.2210/pdb7v63/pdb). Source data are provided with this paper.

Field-specific reporting

Please select the one below that is the best fit for your research. If you are not sure, read the appropriate sections before making your selection.

☒ Life sciences ☐ Behavioural & social sciences ☐ Ecological, evolutionary & environmental sciences

For a reference copy of the document with all sections, see nature.com/documents/nr-reporting-summary-flat.pdf

Life sciences study design

All studies must disclose on these points even when the disclosure is negative.

Sample size	Sample sizes were not predetermined based on statistical methods, but were chosen according to the standards of the field (at least three independent biological replicates for each condition).
Data exclusions	In high content imaging, some images with over-exposure were excluded from calculation
Replication	Reported results were consistently replicated across at least triple experiments with all replicates generating similar results.
Randomization	No human or animal subjects were used in the study. The cells were randomly equally distributed onto 96-well plates in our cellular experiments.
Blinding	Investigators were not blinded. Blinding during collection and analysis was not feasible because data were collected by HCS . Blinding is not typically used in the field.

Reporting for specific materials, systems and methods

We require information from authors about some types of materials, experimental systems and methods used in many studies. Here, indicate whether each material, system or method listed is relevant to your study. If you are not sure if a list item applies to your research, read the appropriate section before selecting a response.

Materials & experimental systems

n/a	Involved in the study	
☐	☒	Antibodies
☐	☒	Eukaryotic cell lines
☒	☐	Palaeontology and archaeology
☒	☐	Animals and other organisms
☒	☐	Human research participants
☒	☐	Clinical data
☒	☐	Dual use research of concern

Methods

n/a	Involved in the study	
☒	☐	ChIP-seq
☒	☐	Flow cytometry
☒	☐	MRI-based neuroimaging

Antibodies

Antibodies used	All antibodies are listed in the Materials section including brand and catalog numbers on page 9 and the Methods section including final concentrations as used in experiments on page 13. Anti-uPAR antibody ATN658 was a kind gift from Dr. Andrew Mazar of Monopar Therapeutics Inc. Horseradish peroxidase-conjugated antibody to rabbit IgG (S0101) or to mouse IgG (S0100) and anti-GAPDH (G0100) were purchased from LabLead; anti-phospho-ERK1/2 (p-ERK1/2) (#4370) was purchased from Cell Signaling Technology; anti-ERK1/2 (T55487) was from Abmart. Polyclonal rabbit anti-human uPAR antibody was prepared by Zoonbio biotechnology company using our recombinant soluble uPAR as antigen.
Validation	Horseradish peroxidase-conjugated antibody to rabbit IgG, LabLead, cat#S0101, western blot analysis of HEK293T cell lysate, using antibody at dilution of 1: 5,000 (see manufacturer's website: http://www.lablead.cn/product/view/17565); horseradish peroxidase-conjugated antibody to mouse IgG, LabLead, cat#S0100, western blot analysis of HEK293T cell lysate, using antibody at dilution of 1: 5,000 (see manufacturer's website: http://www.lablead.cn/product/view/17564); GAPDH(MC4) mouse monoclonal antibody, LabLead, cat#G0100, western blot analysis of HEK293T cell lysate, using antibody at dilution of 1: 3,000 (see manufacturer's website: http://www.lablead.cn/product/view/17258); anti-phospho-ERK1/2 (p-ERK1/2) , Cell Signaling Technology, cat#4370, western blot analysis of HEK293T cell lysate, using antibody at dilution of 1: 1,000 (see manufacturer's website: https://www.cellsignal.cn/products/primary-antibodies/phospho-p44-42-mapk-erk1-2-thr202-tyr204-d13-14-4e-xp-rabbit-mab/4370?site-search-type=Products&N=4294956287&Ntt=4370&fromPage=plp); anti-ERK1/2, Abmart, cat #T55487, western blot analysis of HEK293T cell lysate, using antibody at dilution of 1: 10,000 (see manufacturer's website: http://www.ab-mart.com.cn/page.aspx?node=%20117%20&id=%201727); polyclonal rabbit anti-human uPAR antibody for immunoblotting in each blot with negative control is shown in Fig. 4a and 5c.

Eukaryotic cell lines

Policy information about cell lines

Cell line source(s)	HEK293T cells for virus production and stable cell lines construction have been purchased from ATCC. Drosophila S2 cell was purchased from Invitrogen.
Authentication	Cell lines have been authenticated based on their morphology observed with light microscopy but not by additional methods.
Mycoplasma contamination	Cell lines were tested negative for mycoplasma contamination by One-step Quickcolor Mycoplasma Detection Kit(T102,clark bioscience)
Commonly misidentified lines (See ICLAC register)	No
